# Gene and protein analysis reveals that p53 pathway is functionally inactivated in cytogenetically normal Acute Myeloid Leukemia and Acute Promyelocytic Leukemia

**DOI:** 10.1186/s12920-017-0249-2

**Published:** 2017-03-24

**Authors:** Julia Abramowitz, Tzahi Neuman, Riki Perlman, Dina Ben-Yehuda

**Affiliations:** 1 Department of Hematology, Hadassah-Hebrew University Medical Center, P.O. Box 12000, Jerusalem, 91120 Israel; 2 Department of Pathology, Hadassah-Hebrew University Medical Center, Jerusalem, Israel

## Abstract

**Background:**

Mechanisms that inactivate the p53 pathway in Acute Myeloid Leukemia (AML), other than rare mutations, are still not well understood.

**Methods:**

We performed a bioinformatics study of the p53 pathway function at the gene expression level on our collection of 1153 p53-pathway related genes. Publically available Affymetrix data of 607 de-novo AML patients at diagnosis were analyzed according to the patients cytogenetic, FAB and molecular mutations subtypes. We further investigated the functional status of the p53 pathway in cytogenetically normal AML (CN-AML) and Acute Promyelocytic Leukemia (APL) patients using bioinformatics, Real-Time PCR and immunohistochemistry.

**Results:**

We revealed significant and differential alterations of p53 pathway-related gene expression in most of the AML subtypes. We found that p53 pathway-related gene expression was not correlated with the accepted grouping of AML subtypes such as by cytogenetically-based prognosis, morphological stage or by the type of molecular mutation. Our bioinformatic analysis revealed that p53 is not functional in CN-AML and APL blasts at inducing its most important functional outcomes: cell cycle arrest, apoptosis, DNA repair and oxidative stress defense. We revealed transcriptional downregulation of important p53 acetyltransferases in both CN-AML and APL, accompanied by increased Mdmx protein expression and inadequate Chk2 protein activation.

**Conclusions:**

Our bioinformatic analysis demonstrated that p53 pathway is differentially inactivated in different AML subtypes. Focused gene and protein analysis of p53 pathway in CN-AML and APL patients imply that functional inactivation of p53 protein can be attributed to its impaired acetylation. Our analysis indicates the need in further accurate evaluation of p53 pathway functioning and regulation in distinct subtypes of AML.

**Electronic supplementary material:**

The online version of this article (doi:10.1186/s12920-017-0249-2) contains supplementary material, which is available to authorized users.

## Background

Acute Myeloid Leukemia (AML) is the most common acute leukemia affecting adults with an estimated 18,860 new AML cases in USA alone in 2014 [1]. AML is a heterogeneous disease that can be divided into many subtypes. Three classifications of AML patients are based on cytogenetics (karyotype), the degree of myeloblast maturity (FAB, French-American-British system) or molecular mutations acquired by the myeloblasts. Specific cytogenetic abnormalities can be found in many AML patients and the type of chromosomal abnormality has a prognostic significance [2], as well as the type of molecular mutation [3]. In this work we studied 2 subtypes of AML: the cytogenetically normal AML (CN-AML) and Acute Promyelocytic Leukemia (APL). CN-AML comprises almost half of all adult AML patients and is of intermediate prognosis. APL comprises 5–10% of all AML cases. APL is characterized by a chromosomal translocation t(15;17) that creates the fusion oncogene PML-RARA. APL is of good prognosis and can be treated successfully with high doses of vitamin A (ATRA).

Gene expression profiling (GEP) of ample of genes can create a comprehensive picture of AML pathogenesis [4]. Specifically, there has been an effort to identify genome-wide expression signatures that distinguish between different AML subtypes [5–10] and in particular between different subgroups of CN-AML [5, 11–14]. We used the wealth of GEP data to examine the p53 pathway in AML.

P53 is a multifaceted and omnipotent tumor suppressor and its inactivation is an important requirement for unrestrained growth of tumor cells [15]. Indeed, the p53 gene is mutated in half of all human tumors. However, in hematological malignancies mutant p53 occurs only in 11.1% of the cases according to version R15 of the IARC database [16]. In AML, mutations in the p53 gene were found in 4.5–15% of all cases [17–20] , with less than 2.5% of CN-AML patients [21, 22] and none in APL [23, 24] patients. We also sequenced p53 gene in 22 APL samples and found it to be wt in all (Additional file 1). Mechanisms that allow hematopoietic malignant cells to inactivate the p53 pathway are still mostly elusive. We investigated the functional status and regulation of the p53 pathway in AML, specifically in CN-AML and APL patients.

The tight constraints on p53 are mainly wielded by its negative regulators, Mdm2 and Mdmx. P53 regulates its own intracellular level under normal physiological conditions through an auto-regulatory feedback loop with Mdm2 in which p53 transcribes the Mdm2 gene, while Mdm2 protein ubiquitinates p53 and thus targets it for degradation [25, 26]. Following stress, post-translational modifications of Mdm2 [27], that result in Mdm2 degradation or inhibition, allow activation of p53. During normal hematopoiesis Mdm2 is required to regulate p53 levels and allow stem cell, lymphocyte and myeloid progenitors survival [28, 29].

The other major negative regulator of p53, Mdmx [30], is a structural homolog of Mdm2 that lacks the E3 ligase function. Instead, Mdmx associates with the transcriptional activation domain of p53 and inhibits p53 transcriptional activity by inhibition of p300/CBP-mediated acetylation of p53 [31]. Overexpression of Mdmx was associated with wild-type p53 in the majority of malignancies examined [32–36], suggesting that high levels of Mdmx can inhibit the p53 pathway, substituting for mutations in p53. Several studies demonstrated the significance of Mdmx in the hematopoietic system [37–41].

The functional status of p53 pathway in different subtypes of AML is yet to be revealed. We compared the expression of p53 pathway-related genes in 27 AML subtypes and found differential alterations among them. Although many papers addressed gene expression and protein levels of p53 and Mdm2 in AML, only 1 study tested Mdm2 gene expression specifically in CN-AML and APL [42]. All other p53 regulatory molecules studied in this work were not previously examined in CN-AML or APL. We performed in depth analysis of p53 pathway-related gene and protein expression in CN-AML and APL. We found that p53 is functionally inactivated and suggest that this is probably by inhibition of p53 protein acetylation. The fourth decade of the p53 pathway research brings new p53-based drugs to treat cancer [43]. There is therefore a need in accurate evaluation of p53 pathway functioning and regulation in distinct subtypes of AML that can point to an appropriate therapy for every patient.

## Methods

Detailed information about the methods is available in Additional file 1.

### The parameters for choosing p53 pathway related genes for bioinformatics-

We constructed a list of 1153 genes that are associated with the p53 pathway. It includes genes related to the key components of p53 pathway: p53, Mdm2, Mdmx, Puma, Slug and Chk2. This list is a databases and literature-curated collection of genes for which the association with key components of p53 pathway was biochemically proved by at least one publication.

### Patients and control samples

Bioinformatic analysis of p53 pathway-related gene expression was performed on 4 previously published gene expression arrays of de-novo AML samples at diagnosis [8, 9, 13, 44]. The raw data of these arrays were submitted to the NCBI Gene Expression Omnibus database. The 607 examined AML patients’ samples were provided with clinical data that allowed us to classify patients into 27 AML subtypes by cytogenetics, FAB and molecular mutations parameters. Control group included 74 nonmalignant disorders and normal bone marrow (nBM) samples [10]. The arrays used in this study were conducted on GeneChip® Human Genome U133 Plus 2.0 Affymetrix Array.

### Microarray data analysis

Analysis service was performed by The Center for Cancer Computational Biology, Dana-Farber Cancer Institute, Boston, MA. A linear model was developed to produce gene expression contrasts between leukemic samples and nBM. Using this model, we identified probe sets with significant differential expression (log_2_ Fold Change > 1.5) and multiple comparison adjusted *p*-value < 0.01, using Benjamini and Hochberg method.

A probe set is a collection of probes that identify a specific/single gene. A probe set, rather than a single probe, is used in order to get a better signal for the specific transcript. There may be up to 11 probes in a probe set and sometimes more than one probe set may be used for a single gene. The list of differentially expressed probe sets and differentially expressed genes (DEGs) for each AML subtype are available on demand.

To evaluate whether p53 is active as a transcriptional factor we analyzed p53 pathway-related DEGs by 2 unique approaches. Individual DEGs were classified into functional outcome groups and each gene was placed in the context of p53-dependent activation/repression based on the knowledge from the literature. Additionally, expression of our DEGs was compared to the literature-based gene expression signatures (discussed in the text, raw data is not shown).

### PCR

Real-Time PCR was performed on bone marrow samples of 23 CN-AML and 28 APL patients at diagnosis (Additional file 2). Normal bone marrow samples from Hodgkin’s lymphoma patients without bone marrow involvement served as controls (25 for CN-AML and 34 for APL). All RNA samples were originally collected for clinical needs. Real-Time PCR was performed using ABI TaqMan gene expression assays.

### Immunohistochemistry

Immunohistochemistry was performed on bone marrow samples of 25 CN-AML and 23 APL patients at diagnosis (Additional file 2). Normal bone marrow samples of 35 non-hematological patients (mainly with fever of unknown origin) served as controls. All samples were originally collected for clinical needs.

## Results

### Database of p53 pathway-related genes

We constructed a comprehensive list of genes that are associated with key proteins of the p53 pathway: p53, Mdm2, Mdmx, Chk2 (an upstream activator of p53 in the DNA damage response pathway) [45–47], Puma (pro-apoptotic p53 target gene [48, 49]) and Slug (an anti-apoptotic p53 target gene, a repressor of Puma expression [50, 51]). Our list consists of 1153 genes, 921 are p53-related genes and the rest are related to the key proteins of the pathway mentioned above. This list is a database and literature-curated collection of all genes for which the association with the key proteins of p53 pathway was supported biochemically in at least one publication (up to April 2011). The complete list is presented in Additional file 3. The distribution of the genes between key proteins of p53 pathway and the overlap between them is presented in Additional file 4. The 1153 genes were categorized according to the functional outcomes of p53 protein and factors that regulate p53 pathway (Table 1).

**Table 1:** Functional distribution of 1153 p53-related genes analyzed by bioinformatics

	Number of genes
P53-related functional outcomes-	
apoptosis	131
cell cycle	103
DNA repair	49
oxidative stress	25
metabolism	58
nervous system	17
cytokines and inflammation	36
cytoskeleton/structural	41
extracellular matrix	29
senescence	17
hypoxia	9
nuclear receptors	8
localization	4
other	84
Regulation of p53 by	
transcription machinery/translation	110
transcriptional activators	53
transcriptional repressors	36
chaperons	13
ubiquitination	48
deubiquitination	7
phosphorylation	21
dephosphorylation	8
acetylation	16
deacetylation	20
methylation	8
sumoylation	14
neddylation	3
isomerization	3
Other pathways related to p53	138
KEGG	44
Total	1153

The genes were categorized by functional outcomes of p53 pathway and factors that regulate it

### Bioinformatic analysis of AML subtypes

Bioinformatic analysis of the p53 pathway was based on publicly available data from Affymetrix gene expression arrays performed on 607 AML samples and 74 nBM controls. AML is a heterogeneous disease that can be divided into many subtypes based on various criteria. We grouped the 607 AML patients into 11 subtypes by cytogenetics, 8 subtypes by FAB or 8 subtypes by molecular mutations (Table 2). The expression levels of 1153 p53 pathway-related genes were compared between AML patients of each subtype and normal controls using a linear model that was developed for this purpose and statistically significant differentially expressed genes (DEGs) were identified. For number of DEGs identified in each AML subtype see Table 2. All the examined groups showed differential gene expression between leukemia patients and controls. This result indicates that the p53 pathway is altered in AML in comparison to nBM.

**Table 2:** The number of analyzed patients and revealed p53-related DEGs in 27 AML subtypes

AML subtypes	Number of patients included in analysis	Number of p53-related DEGs
Cytogenetic groups		
Normal	290	147^*^
t (15;17)	34	172^*^
t (8;21)	39	202^*^
inv (16)	37	147^*^
11q23	10	165^*^
-5/7 (q)	31	102^*^
trisomy 8	26	109^*^
Complex	23	104^*^
t (6;9)	5	176^*^
t (9;22)	3	78
-9(q)	7	88
FAB groups		
M0	48	145^*^
M1	136	90^*^
M2	157	37
M3	34	172^*^
M4	108	39
M4E	5	185^*^
M5	110	68^*^
M6	9	42
Molecular mutations		
FLT3-ITD	132	128^*^
FLT3-TKD	46	64
NPM1	154	151^*^
CEBPA	33	97
NRAS	44	143^*^
KRAS	4	171^*^
EV1	28	163^*^
RUNX1	11	143^*^
Total number of patients	607	

^*^significant enrichment (*p*-value <0.01) for p53 pathway-related genes

To assess the specific enrichment of p53 pathway-related genes, we performed bootstrapping procedure that compared the p53 pathway related DEGs to the distribution of 1153 randomly selected genes within the chip array. Significant over-representation of p53 pathway-related DEGs (p < 0.01) was found in the majority of AML samples when subtyped by cytogenetics, 5 out of 8 FAB subtypes and in 6 molecular mutation subtypes (Table 2). This significant enrichment of p53 pathway-related DEGs in the majority of AML subtypes signifies that the alterations in p53 pathway play a role in leukomogenesis.

Unsupervised hierarchical clustering of all AML cytogenetic subtypes based on their DEGs values (Additional file 5A) showed that p53 pathway-related gene expression was not correlated with prognostic status of cytogenetic subtypes. It was neither correlated with morphological stage of blasts (by FAB) or with type of their molecular mutation (Additional file 5B, C). This indicates the need to separately examine each AML subtype for alterations in the p53 pathway, rather than grouping by the accepted classifications.

**Fig. 1:**
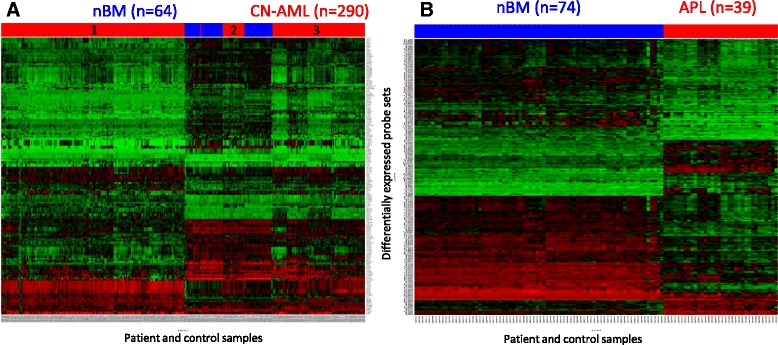
Heat maps of differentially expressed p53-related probe sets in CN-AML and APL. **a** CN-AML, **b** APL. Red -upregulation of gene expression, green-downregulation of gene expression, black-no change in gene expression. Numbers of enrolled patients (*red*) and controls (*blue*) are indicated on top and color coded. Subgroups of CN-AML patients are designated as 1, 2 and 3

The rest of this study is focused on CN-AML and APL patients. The list of p53 pathway-related DEGs in CN-AML and APL is presented in Additional file 6. Differentially expressed probe sets in patients and controls were graphically illustrated in a clustered heat map (Fig. 1). In previous studies CN-AML patients were divided into subgroups by overall gene expression profiling [5, 6, 11–14]. Interestingly, we found that CN-AML patients were divided into 3 subgroups also by the p53 pathway-related gene expression profiling (Fig. 1 and Additional file 5A). As expected, APL patients displayed homogeneous pattern of gene expression [52]. Despite the differences between the 2 leukemias, our bioinformatic analysis revealed that 41.6% of DEGs were common to both CN-AML and APL blasts and their expression was correspondingly upregulated or downregulated in comparison to nBM (Additional file 7).

### Analysis of the p53 pathway in CN-AML and APL

To evaluate whether p53 is active as a transcriptional factor in CN-AML and APL patients we analyzed the p53 pathway-related DEGs (Additional file 6) by 2 approaches: individual DEGs and literature-based gene expression signatures.

DEGs were classified into functional outcome groups (Table 1). Herein we present the most important ones: cell cycle, apoptosis, DNA repair and oxidative stress defense.

#### Genes associated with cell cycle

**Fig. 2:**
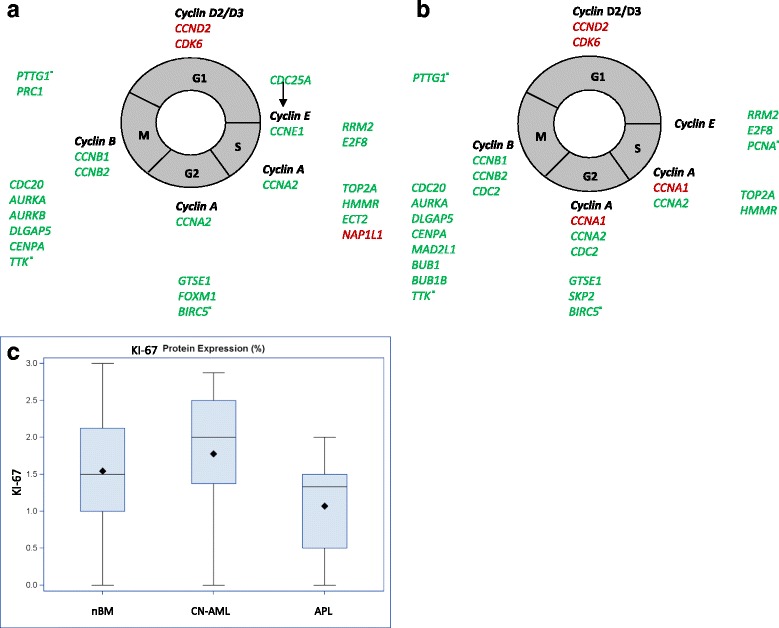
Cell cycle in CN-AML and APL. **a**, **b** DEGs that are typically repressed by p53 and are associated with the cell cycle in CN-AML (**a**) and APL (**b**). Canonical cyclins in each phase of the cell cycle are in bold black. DEGs implicated in the different phases of the cell cycle are placed near the appropriate phase. Upregulated DEGs are in red and downregulated DEGs are in green. Most of the DEGs arrest proliferation, while only CCND2, CDK6 and NAP1L1 genes support proliferation. Most of the genes originate from 2 gene expression signatures [71, 72], Additional 13 genes are marked withˣ. CCNE1 (**a**) is the only DEG not repressed by p53. **c** Percent of KI-67 positive cells. Immunohistochemistry was performed on bone marrow samples of 25 CN-AML and 23 APL patients at diagnosis and 35 nBM samples. The boundaries of the blue box indicate the 25^th^ percentile (bottom boundary) and the 75^th^ percentile (top boundary) of KI-67 level, median is displayed by the thick line in the box, mean by rhombus sign

One of the most important functional outcomes of the activation of the p53 pathway is cell cycle arrest. The list of cell cycle-related DEGs in CN-AML and APL is available in Additional file 8. First, we positioned cell cycle-related DEGs to the cell cycle phases. In both CN-AML and APL there was an increase in the expression of genes essential for progression through the G1 phase, such as CDK6 and Cyclin D2. However, in both CN-AML and APL there was also downregulation of about 20 cell cycle promoting genes, including Cyclin E1 in CN-AML, Cyclins A2, B1 and B2 in both CN-AML and APL and CDC2 in APL (Fig. 2a and b). Cyclin A1was upregulated in APL, in agreement with published results [53]. Interestingly, cyclin A1 was shown to induce cell arrest and apoptosis in carcinoma cells [54]. Overall, the upregulation of G1 cell cycle genes together with the downregulation of other cell cycle promoting genes including essential cyclins and CDC2 beyond the G1 phase indicate that AML blast cells in these two sub-types do not proceed in cell cycle, but accumulate in the G1 phase. To confirm these results we examined the proliferation in BM biopsies of CN-AML and APL patients diagnosed in our hematology department by the proliferation marker KI-67. IHC demonstrated that the percentage of KI-67+ cells were not significantly different between normal bone marrow (20%) and either APL (<15%) or CN-AML (25%) biopsies (Fig. 2c and Additional file 9). This result is in agreement with low white blood cell count characteristic to APL patients. Yet, CN-AML patients are characterized by increased white blood cell count (Additional file 2 and [55]). Indeed, we found a significant positive correlation between KI-67 and white blood cell (WBC) count in CN-AML patients (correlation coefficient 0.59, *p*-value 0.0016) suggesting that the slight increase of KI-67 is associated with increased WBC count. The notion that CN-AML and APL are not highly proliferative malignancies is not well recognized despite the agreement with previous studies [56–67] and should be explored further.

Next, we evaluated the transcriptional activity of p53 in leukemic cells. None of the canonical p53-induced cell cycle arrest genes (p21 [68], 14-3-3 [69], reprimo and mcg10 [70]) were DEGs in our bioinformatic analysis (Additional file 8), while cell cycle arrest gene Gadd45 was downregulated in APL. With regards to p53-dependent cell cycle arrest by transcriptional repression, a panel of 69 cell cycle regulatory genes subjected to p53-dependent transcriptional repression following DNA damage was identified [71, 72]. We examined 40 of these genes and found that 17 were repressed DEGs in each of CN-AML and APL subtypes. These 17genes, as well as additional downregulated cell cycle promoting genes are depicted in Fig. 2a and b. Importantly, it has been demonstrated that the pattern of activated p53 includes induction of cell cycle arrest genes together with the repression of cell cycle promoting genes [73, 74]. While we observed repression of cell cycle regulatory genes, our bioinformatics analysis did not present induction of the most important cell cycle arrest genes in CN-AML and APL samples. Taken together, our results suggest impaired transcriptional activity of p53 probably resulting in impaired p53-dependent cell cycle arrest.

#### Genes associated with apoptosis

Another important functional outcome of the activation of the p53 pathway is initiation of apoptosis by transcriptional induction of pro-apoptotic genes [75], transcriptional repression of anti-apoptotic genes [76] or via its well established transcriptional-independent role [77]. The list of apoptosis-related DEGs is available in Additional file 10. First, we analyzed DEGs that are related to the Bcl2 family and found a significant upregulation of the anti-apoptotic Bcl-2 and Bcl-xL genes in CN-AML and APL respectively, accompanied by a downregulation of proapoptotic genes, Bid in both subtypes and Bik and Bim in CN-AML. The only pro-apoptotic gene to be upregulated in CN-AML was Noxa gene. Due to these results we conclude that the Bcl2 family does not induce apoptosis in either CN-AML or APL.

**Fig. 3:**
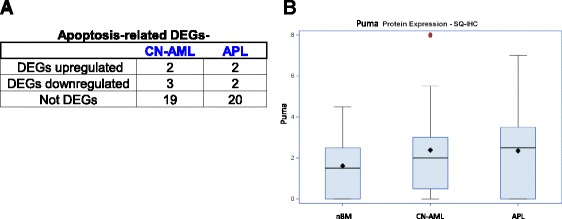
DEGs associated with apoptosis. **a** Apoptosis-related DEGs in CN-AML and APL among canonical p53-dependent pro-apoptotic genes [75]. We extended some gene families (e.g. PIGs) and the resultant gene collection includes 24 genes. The table summarizes the numbers of genes that were found to be upregulated DEGs, downregulated DEGs or not identified as DEGs in our study. **b** Puma protein levels by IHC in nBM, and in CN-AML and APL patients’ BM. Symbols of box plots are as in Fig. 2; outlying value is marked by red circle; tails of the distribution depicted only in one direction indicate that the values are skewed towards that side of the average

In addition, our analysis of a collection of 24 canonical p53-dependent pro-apoptotic genes [75] demonstrated that the expression of most of the genes (19/24 and 20/24) was unaltered in both CN-AML and APL patients (Fig. 3a).

Potential induced and repressed targets of p53 during genotoxic stress-induced apoptosis were identified by Kho et al. [78]. Of 38 genotoxic stress-induced genes 31 were included in our p53 pathway-related list. Only 1 of these 31 genes, was induced in CN-AML and none in APL indicating that there was no induction of p53-dependent apoptotic genes in the examined leukemias. Of the 175 genotoxic stress-repressed genes [78] 38 were included in our p53 pathway-related list. Of these, 10 were downregulated in both CN-AML and APL, however only 1 of the downregulated genes, KLF5, was apoptosis-related, while the rest 9/10 were not apoptosis-related. Taken together, the bioinformatic analysis indicates that p53-dependent apoptosis is not activated in CN-AML and APL.

Lastly, Puma is a potent upstream regulator and downstream mediator of p53-dependent apoptosis in hematopoietic cells [79–81]. We examined Puma protein levels by IHC and found that it was not upregulated in leukemic cells (Fig. 3b, mainly by SQ-IHC score). This is in agreement with the absence of p53-dependent transcriptional induction of apoptosis in CN-AML and APL leukemias revealed by the bioinformatics.

#### Genes associated with DNA repair

P53 participates in the modulation of DNA repair and recombination through both transcriptional-dependent and independent functions. In our bioinformatics analysis most DNA repair-related genes were downregulated in both leukemias, including canonical p53 transcriptional targets important for DNA repair, like RRM2 (Additional file 11). Of 91 literature-based DNA repair-related genes [52, 71, 82] 43 were included in our p53 pathway-related list, but none was upregulated and 2 were downregulated in both leukemias. Therefore we conclude that there was no induction of p53-related DNA repair in CN-AML and APL.

#### Genes associated with oxidative stress defense

Under mild levels of reactive oxygen species (ROS) p53 is expected to induce the transcription of anti-oxidant genes [83, 84]. However p53 did not induce any of its 7 anti-oxidant target genes in our analysis (Additional file 12). This result suggests that there is no p53-dependent anti-oxidant defense in CN-AML and APL. High levels of ROS lead to oxidative stress which induces a p53-dependent transcription of pro-oxidant and pro-apoptotic genes facilitating apoptotic cell death [83, 84]. Our bioinformatic analysis showed that only 1 of 4 pro-oxidant genes was induced in APL and none in CN-AML. Moreover, bioinformatic analysis of a further 9 p53 target genes identified to be upregulated during oxidative stress [85] showed no change in expression in the CN-AML and APL patient cells. Taken together, the above analyzes indicate no induction of p53-dependent oxidative stress defense.

### Literature-based gene expression signatures

We compared the expression of our DEGs to literature-based gene expression signatures that correspond to DNA damage-induced response enriched for p53 pathway genes [71–74, 78, 85–90]. The analysis of several literature-based signatures related to cell cycle, apoptosis and oxidative stress defense [71–74, 78, 85] was presented above. Analysis of additional 5 signatures is summarized in Table 3. Only a few signature genes were upregulated or downregulated as expected by the signature, while several others were expressed in the opposite direction. The expression of the majority (78%) of examined p53-related genes was unaltered (119/152). Thus we conclude that there is no activation of the p53 pathway in CN-AML and APL.

**Table 3:** Comparison of our bioinformatics results to literature-based gene expression signatures of DNA damage-induced response

Ref	System	Literature-based gene signature		Our bionformatic results	
		Number of genes altered in the signature	Number of genes included in our p53 list	DEGs in CN-AML	DEGs in APL
[85]	p53-dependent transcriptional response of cell lines to 9 DNA-damaging agents	16 ↑	12↑	0 ↑ 1 ↓	0↑ 1 ↓
[86]	transcriptional response of human cells to ionizing radiation	199 ↑ 49 ↓	59 ↑ 11 ↓	1↑ 6↓	1↑ 7↓
[87]	healthy PB cells irradiated ex vivo	61^a^	29	3	5
[88]	34 patients before and after irradiation^b^	23^a^	12	1	1
[89]	7 AML patients before and after chemotherapy^c^	30↑ [113 ↑]^d^	29↑	1↑ 2↓	0↑ 3↓
	Total		152	33/152 DEGs, 119/152 not DEGs	

We compare how many of upregulated/downregulated genes in the signature are indeed upregulated or downregulated DEGs in our analysis of CN-AML and APL, ↑- upregulated gene expression, ↓- downregulated gene expression

^a^Only the list of the signature genes was provided, without the specific names of up- and down-regulated genes. Therefore, we present the overall number of upregulated and downregulated DEGs

^b^All are hematological patients

^c^5 are CN-AML patients and 1 is APL

^d^30 of the upregulated 113 genes are associated with p53 regulation

### Regulation of the p53 pathway in CN-AML and APL

So far our bioinformatic analysis of the p53 pathway in CN-AML and APL indicates that p53 protein is inactive in leukemia. We next investigated the mechanism responsible for p53 inactivation. We examined proteins that regulate p53 transcription, stability and posttranslational modifications both at the mRNA level, using bioinformatics and Real-Time PCR tools, and at the protein level using IHC.

#### Expression levels of genes regulating p53

The regulation of p53 is exerted mostly at the protein level, yet assessment of this regulation at the gene expression level might shed light on this process. Our bioinformatic analysis showed upregulated expression of p53 in both CN-AML and APL (Additional file 6). Indeed, there were more DEGs that allow increased transcription of p53 than those that indicate the opposite (Additional file 13). The co-activators and co-repressors DEGs of p53 transcriptional activity are presented in Additional file 14, but their contribution is not clear.

Control of the levels of p53 protein occurs via ubiquitin-dependent degradation through proteasome [91]. None of the 14 most known E3 ligases [91, 92] present in our p53-related list were DEGs either in CN-AML or APL patient samples (Additional file 15). Also, the number of DEGs that indicate increased stability of p53 protein was higher than the number of genes that indicate the opposite, especially in CN-AML. Taken together, these results imply that the regulation of the p53 protein stability is not impaired in the examined leukemic groups.

The expression of genes implicated in post-translational regulation of p53 is presented in Additional file 16. Our bioinformatics analysis showed almost no DEGs related to methylation and sumoylation of p53, along with the inconclusive impact of phopshorylation-related DEGs. Acetylation of p53 protein is essential for its transcriptional function [93, 94]. Interestingly, we found downregulation of p300 and P300/CBP-associated factor (PCAF), important 53-acetylating acetyltransferases, in both CN-AML and APL. Importantly, we detected a significant 4 fold downregulation of the PCAF gene in our CN-AML and APL patients (p < 0.01) by Real-Time PCR (data not shown). Additionally, CARM1 (coactivator-associated arginine methyltransferase 1), which has a positive cooperative effect with p300 on p53-dependent transcription [95], was also downregulated in both CN-AML and APL. Mdmx, a key negative regulator of p53, inhibits p53 transcriptional activity by inhibition of p300/CBP-mediated acetylation of p53 [31]. Mdmx expression was increased in both CN-AML and APL in our bioinformatic analysis (Additional file 6-Mdm4) although only by 1 out of 7 array probe sets. Nonetheless, we found significant upregulation of Mdmx in CN-AML patients (1.72 fold, p-value < 0.01) by Real-Time PCR analysis. Taken together, the acetylating pathway that activates p53 showed overall impairment and thus p300, PCAF, CARM-1 and Mdmx, are promising candidates to play a role in deregulation of the p53 pathway in leukemia.

#### Protein levels of key p53 pathway components

The protein levels of key p53 pathway proteins (p53, Mdm2, Mdmx and Chk2) were evaluated by immunohistochemistry (IHC) staining (Additional file 9). IHC results are interpreted by a SQ score (semi-quantitative) that is a multiplication of percent stained cells and intensity of the staining (Additional file 17). Intensity of staining allows examining the level of protein at the single cell level, whereas SQ score considers the total cell population, similarly to western blot analysis.

**Fig. 4:**
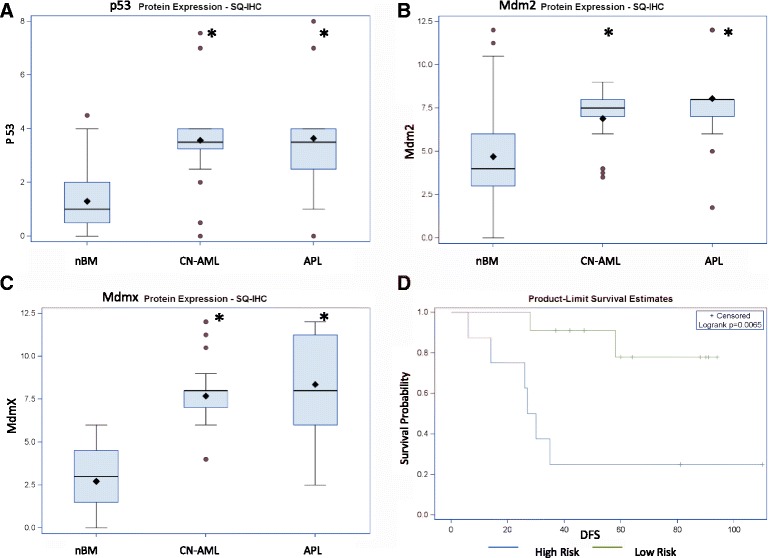
p53, Mdm2 and Mdmx protein levels in nBM, CN-AML and APL. **a** P53 protein, **b** Mdm2 protein, **c** Mdmx protein; protein levels are according to IHC ( SQ score); *-p-value < 0.05 (compared to nBM); Box plots symbols are as in Fig. 2 and 3. **d** Kaplan-Meier survival curve of APL patients according to Mdmx protein levels. Mdmx high risk group (*blue*) n = 8, low risk (*green*) n = 11. DFS- disease free survival

Non-functional p53 pathway can be a result of inadequate levels of p53 protein. However we observed significantly increased p53 levels in BM of both CN-AML and APL vs nBM (SQ score Means 3.57, 3.64 and 1.30, respectively) (Fig. 4a). This increase was a result of a significantly higher fraction of leukemic cells expressing p53 (75%) compared to nBM (10%). Notably however, the intensity of p53 protein was similarly low in both normal and leukemic BM, especially in comparison to several occasional cells exhibiting high level of p53 (Additional files 9 and 17).

Mdm2 is a well known regulator of the p53 protein levels. We observed a significant increase of Mdm2 SQ score in both CN-AML and APL vs nBM (Means 6.89, 8.04 and 4.68, respectively) (Fig. 4b). This increase was a result of a significantly higher fraction of leukemic cells expressing Mdm2 (93%) compared to nBM (30%). The intensity of Mdm2 staining was (however) not elevated in leukemic blasts.

High levels of Mdmx protein can explain a non-functional status of p53 protein as a transcription factor. Indeed, we revealed an increased Mdmx SQ score in both CN-AML and APL (Means 7.68, 8.36, respectively) vs nBM (2.71) (Fig. 4c). The fraction of cells expressing Mdmx protein in CN-AML and APL (75%) was significantly increased compared to nBM (20%). In addition, the intensity of Mdmx staining was also significantly increased by 37% and 50% in APL and CN-AML, respectively (Additional file 17).

Importantly, we found a significant association (logrank p < 0.01) between the Mdmx SQ score and disease free survival (DFS) of APL patients. The Kaplan-Meier curve (Fig. 4d) shows that patients with high levels of Mdmx (SQ ≥ 9) comprise a high risk group with sooner and more occurrences of relapse compared to patients with lower levels of Mdmx (SQ <9) forming a low risk group.

In the previous years it was common to estimate the percent of patients that overexpress the examined protein. Our analysis showed that p53, Mdm2 and Mdmx proteins were overexpressed in more than 60% of CN-AML or APL patients compared to percentile 90 of nBM expression (Additional file 18).

**Fig. 5:**
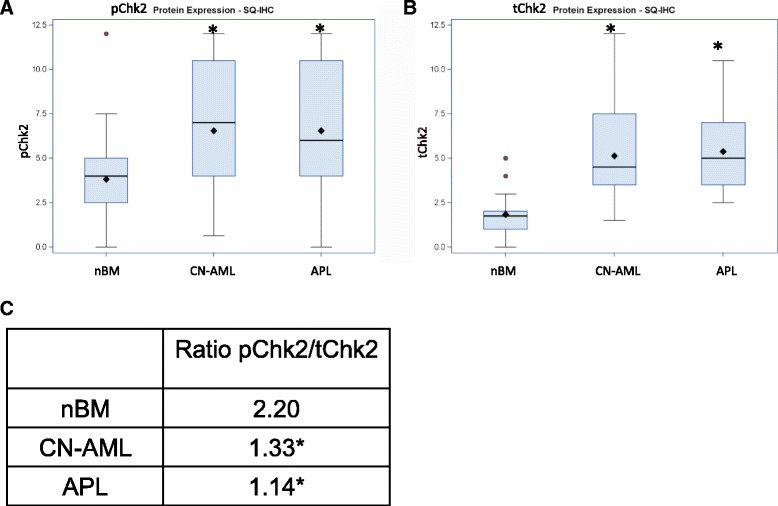
pChk2 and tChk2 protein levels in nBM, CN-AML and APL. **a** pChk2 protein, **b** tChk2 protein levels by SQ score. Box plots symbols are as in Fig. 2 and 3. **c** ratio of pChk2 to tChk2 by the median SQ score. *-*p*-value < 0.05 (compared to normal bone marrow)

Chk2 is an immediate upstream activator of p53 during DNA damage response since Chk2-mediated phosphorylation of p53 promotes the association of p53 with p300 and positively regulates its transcriptional activity [46, 96–98]. Therefore, in order to evaluate upstream activation of p53, we examined the protein levels of Chk2 (total, tChk2) and activated Chk2 (phosphorylated on Thr68, pChk2). We observed a significant SQ increase of both tChk2 and pChk2 levels in both CN-AML and APL (Fig. 5a and b). Importantly, while the ratio of pChk2 to tChk2 (median SQs) in nBM was 2.2, it was only1.3 in CN-AML and 1.1 in APL (Fig. 5c). A similar trend of decreased pChk2/tChk2 ratio in leukemia was observed when calculated by percentage of stained cells or by intensity. The decrease of the pChk2/tChk2 ratio in leukemias was statistically significant and points to reduced activation of Chk2 in leukemias. This is in line with previously published research regarding low levels of Chk2 activation in AML [99, 100] and APL [101].

## Discussion

The wealth of the gene expression data allows examining molecular signaling pathways at the gene expression level. The focus of this study was the p53 pathway. Mutations in p53 gene are rare in AML leading to the assumption that the p53 pathway is inactivated by alternative mechanisms. We investigated the functional status and the regulation of the p53 pathway in different AML subtypes, particularly in patients with CN-AML and APL. We first constructed a comprehensive list of 1153 p53 pathway-related genes which is to the best of our knowledge, the most comprehensive record of p53 pathway-related genes, updated to April 2011 (Additional file 3). This list can contribute to further multifaceted research in the field of p53.

We detected significant enrichment of p53 pathway-related DEGs, above the genomic background, in most of the AML subtypes (Table 2). This finding illustrates that changes in p53 pathway play a role in AML leukomogenesis. We found that p53 pathway-related gene expression was not correlated with the accepted grouping of AML subtypes such as by cytogenetically-based prognosis, morphological stage or by the type of molecular mutation (Additional file 5). Interestingly, Haferlach et al. [10] demonstrated similar results of whole genome expression in prognostically different AML cytogenetic subtypes. Our findings signify that analysis of the p53 pathway should not be performed on grouped AML subtypes, but rather separately for each subtype. Thus, in this study we investigated 2 cytogenetic subtypes of AML, CN-AML and APL.

Endogenous DNA damage and defective DNA repair in AML blasts [99, 100, 102–110] should activate the p53 pathway. However, our bioinformatic analysis revealed that p53 is not functional as an activating transcription factor in CN-AML and APL blasts as we did not find induction of genes related to various p53-functional outcomes, cell cycle arrest, apoptosis, DNA repair and oxidative stress defense (Additional files 8, 10, 11, 12). We observed repression of several p53-target genes (mostly related to cell cycle) however this by itself does not indicate p53 activation since the pattern of activated p53 includes both induction and repression of target genes [73, 74, 76, 78]. Analysis of literature-based gene expression signatures further indicated that p53 is transcriptionally non-functional in CN-AML and APL blasts (Table 3). These results are in agreement with the previously observed repression of p53 transcriptional activity in APL mice [111]. Taken together, our results show that p53 is transcriptionally inactive in APL and CN-AML patients.

### Negative regulation of p53 pathway-

Functional inhibition of p53 can be initiated at various levels of its regulation. Our bioinformatic analysis revealed that p53 expression is not inhibited at the transcriptional level (Additional file 13) and that p53 protein stability is probably also not impaired (Additional file 15) in the examined leukemic groups. Percent of AML patients expressing p53 protein is a matter of controversy in the literature [111–117] [20, 24, 118–121]. We found that p53 was overexpressed in more than 60% of CN-AML and APL patient samples compared to normal BM samples (Additional file 18) and it was expressed in approximately 75% of the leukemic cells in the BM sample (Additional file 17A). Yet, p53 level was previously shown to be low to moderate in AML cells [114, 116, 122], with heterogeneity in different AML subtypes [123, 124]. We found low p53 staining intensity in CN-AML and APL blasts, similar to that of nBM (Additional file 17B), in accord with an inactivated p53 pathway. Low levels of p53 in CN-AML were also found by Kornblau et al. [124].

Mdm2 is a well-known negative regulator of p53 protein levels. Previous studies reported overexpression of the Mdm2 gene in AML [42, 123, 125–127]. In contrast, our bioinformatic analysis did not demonstrate overexpression of the Mdm2 gene in APL, CN-AML (Additional file 6) or any other examined AML subtypes (data not shown). Our Real-Time PCR results also demonstrated that there was no significant increase of Mdm2 gene expression in APL and CN-AML patients (data not shown). This result is consistent with an inhibited p53-dependent transcription, since Mdm2 is one of the most important p53 target genes [128, 129]. Regarding Mdm2 protein levels, it was previously demonstrated to be increased in 47-82% of AML patients [42, 130–132], with the need for examining different AML patient subtypes separately as stressed by Faderl et al. [130]. Consistent with this, we found that Mdm2 was overexpressed in 60% of CN-AML and 74% of APL patient samples compared to normal BM samples (Additional file 18). Mdm2 was also significantly increased in the leukemic BM sample by the percentage of stained cells and consequently SQ score (Fig. 4b), consistent with the present dogma of Mdm2 protein overexpression in AML. Increased Mdm2 protein may lead to low levels of p53 protein. Mdm2 can also inhibit p53 by other mechanisms such as directly interfering with recruitment of the acetyltransferases to p53 protein [133, 134]. On the other hand, the intensity of Mdm2 staining was not significantly increased in leukemic cells, questioning Mdm2-dependent p53 inhibition at the level of single CN-AML and APL cells.

A possible mechanism for p53 pathway inactivation is inhibition of p53 transcriptional activity by Mdmx [31, 32]. Indeed, our Real-Time PCR analysis revealed upregulation of Mdmx gene expression in CN-AML, though bioinformatic result was inconclusive. We also found significantly increased Mdmx protein levels in CN-AML and APL leukemia cells compared to nBM by both the percentage of Mdmx-positive cells as well as the intensity of staining (Fig. 4c and Additional file 17). This is in agreement with inhibition of p53 transcriptional activity in CN-AML and APL as was demonstrated by our various bioinformatic analyzes. Importantly, we found that the increased level of Mdmx protein is correlated with higher occurrences of relapse and shorter disease free survival time in APL patients (Fig. 4d). This result positions Mdmx protein as an important inhibitor of the p53 pathway especially in APL as well as in CN-AML. Our findings are in line with reports that revealed upregulated Mdmx protein in various human malignancies [32–35, 37, 38, 40] including AML with complex karyotype [41] and showed the ability of Mdmx to inhibit p53 activity in AML cell lines [135]. Here we present evidence for the link between Mdmx levels and functional inhibition of p53 in CN-AML and APL patients.

High level of Mdmx may impede p53 transcriptional activity by inhibition of p53 acetylation [31] that is essential for its activation [93, 94]. Additionally, our bioinformatic analysis demonstrated downregulation of the important acetylation genes: PCAF (downregulation of PCAF was shown also by Real-Time PCR), p300 and CARM-1 in both CN-AML and APL (Additional file 16). High level of Mdm2 may also contribute to inhibition of p53 acetylation [133, 134] and we indeed found overexpression of Mdm2 in our patients. Inadequate Chk2 protein activation (Fig. 5c) may also be responsible for the impaired acetylation of p53 by p300 [46]. Taken together our data suggest that impaired acetylation of p53 may play an important role in functional inhibition of p53 in CN-AML and APL. These routs of impaired acetylation of p53 protein can accompany p53 deacetylation by HDAC, as it was demonstrated in APL mouse models as a result of p53 deacetylation by HDAC [111]. Our proposition can be evaluated further by examining the effect of acetylation-related therapeutic strategies in CN-AML and APL cells, such as Mdmx inhibitors [136, 137], dual inhibitors of Mdm2 and Mdmx [138, 139] , and their combination with HDAC inhibitors [140]. Mdm2 inhibitor Nutlin-3 might be beneficial since it also enhances acetylation of p53 [141].

Interestingly, bioinformatics analysis revealed a 41.6% similarity between the p53-related gene expression profiles of CN-AML and APL blasts in comparison to nBM (Additional file 7). Analysis of p53 functional outcome genes gave similar results in both groups. Similarly, acetylation-related genes demonstrated analogous expression in both leukemias. Lastly, protein levels of p53 regulators were also parallel in CN-AML and APL BM in comparison to nBM (Fig. 4, Additional file 17). Thus we conclude that CN-AML and APL have a similar pattern of p53 pathway inhibition in comparison to nBM, albeit a different underlying molecular etiology of these diseases.

## Conclusions

We constructed the list of 1153 p53 pathway-related genes. Bioinformatic analysis based on this gene collection demonstrated that p53 pathway is differentially inactivated in different AML subtypes. In depth bioinformatics analysis of the p53 pathway in CN-AML and APL subtypes revealed functional inactivation of p53 protein. Further gene and protein analysis suggested that this may be attributed to impaired acetylation of p53. Our results position Mdmx protein as an important inhibitor of the p53 pathway particularly in APL as well as in CN-AML patients.

## Additional files

Additional file 1: Additional Materials and Methods. (DOCX 63 kb)

Additional file 2: Clinical data of the patients examined in our study by PCR and IHC. (DOCX 13 kb)

Additional file 3: The list of 1153 p53 pathway related genes. (XLS 308 kb)

Additional file 4: Distribution of the genes between key proteins of p53 pathway and the overlap between them. (PPT 122 kb)

Additional file 5: Unsupervised hierarchical clustering of different AML subtypes based on their DEGs values. (PPT 599 kb)

Additional file 6: The list of p53 pathway-related DEGs in CN-AML and APL. (XLSX 40 kb)

Additional file 7: Comparison of DEGs between CN-AML and APL. (XLS 39 kb)

Additional file 8: DEGs related to cell cycle. (XLS 54 kb)

Additional file 9: Examples of IHC staining. (PPT 2209 kb)

Additional file 10: DEGs related to apoptosis. (XLS 54 kb)

Additional file 11: DEGs related to DNA repair. (XLS 32 kb)

Additional file 12: DEGs related oxidative stress. (XLS 33 kb)

Additional file 13: DEGs related to transcription of p53 gene. (XLS 31 kb)

Additional file 14: DEGs related to transcriptional functioning of p53. (XLS 37 kb)

Additional file 15: DEGs related to stability of p53 protein. (XLS 36 kb)

Additional file 16: DEGs related to post-translational regulation of p53. (XLS 37 kb)

Additional file 17: Protein levels by % and intensity. (PPT 394 kb)

Additional file 18: % of patients with protein levels above percentile 90 of nBM. (PPT 143 kb)

## Abbreviations

AML: Acute Myeloid Leukemia
CN-AML: Cytogenetically normal AML
APL: Acute Promyelocytic Leukemia
FAB: French-American-British system
GEP: Gene expression profiling
DEGs: Differentially expressed genes
IHC: Immunohistochemistry
FUO: Fever of unknown origin
SQ: Semi-quantitative score
RFS: Relapse free survival
