# Supplementary material for: Gene and protein analysis reveals that p53 pathway is functionally inactivated in cytogenetically normal Acute Myeloid Leukemia and Acute Promyelocytic Leukemia
Source: BMC Med Genomics. 2017 Mar 24;10:18. doi: 10.1186/s12920-017-0249-2 (PMC5423421; doi:10.1186/s12920-017-0249-2)
Supplement: Supplementary file 1 — Additional Materials and Methods. (DOCX 63 kb) [file 12920_2017_249_MOESM1_ESM.docx]

**Additional Materials and Methods-**

1. **Bioinformatic Methods-**

**The parameters for choosing p53 pathway related genes for bioinformatics-**

This list contains genes that are associated with p53 pathway, while the core of the list consists of genes related to p53 itself. This list is a databases and literature-curated collection of all genes meeting inclusion criteria and it is not hypothesis driven. Some of the genes were selected from the p53 Knowledgebase and IPA p53 Gene View which are the most complete resources for p53 interactions. Additional genes were selected from numerous protein-protein interactions databases (BioGrid, PID, HPRD, MINT, STRING, BIND, UniProt and DIP). Examination of p53 pathways in curated databases like KEGG, Nature Pathway interaction database and Millipore yielded additional selections. Some genes were adopted from array studies of p53 pathway [[1-7](#_ENREF_1)]. Another from comprehensive reviews that depict a variety of p53 functions [[8-14](#_ENREF_8)]. The rest of the genes were selected from the individual research articles using PubMed.

The inclusion criteria for our gene list:

- All included genes are supported by at least one publication that biochemically proved the association between this gene and p53. This is true also for genes obtained from previously published high throughput array studies related to p53 pathway, while the search for a supportive publication was done in PubMed.
- In order to prevent misidentification, in the case of a problematic report of the gene name, verification was done against all the available information published in the research (used antibodies, cited references, function and aliases).
- This list does not contain microRNA genes.

In addition to p53-related genes we attempted to understand the regulation of 5 other molecules that are key components of p53 pathway. These are p53 negative regulators Mdm2 and Mdmx, p53 pathway apoptosis executer Puma, inhibitor of Puma in hematopoietic progenitors Slug and p53 activator subsequent to DNA damage Chk2. For each of our 5 molecules of interest we generated a list of associated genes similarly to the p53 associated gene list. All the sources for the p53- related gene list and all the inclusion criteria were also used to generate these additional 5 lists. The precise numbers of genes in each group and interconnections between them are presented in Additional file 4.

Here we present the combined list of p53, Mdm2, Mdmx, Puma, Slug and Chk2 -related genes which contains 1153 associated genes. All the genes in the list are reference sequences of Homo Sapience, even when the study was performed in other species. For each gene we provide its NCBI gene ID number, official symbol, name and other aliases. For each associated gene in the list we marked whom does it interrelate to, while association is marked as 1 (without differential ranking for more or less verified interactions) and the absence of interaction is not marked at any way. References for each interaction are provided as NCBI PMID. KEGG (Kyoto Encyclopedia of Genes and Genomes) group represents genes that were found to be related to p53 pathway albeit not directly. We included these genes in our analysis in order to understand better the general context of p53 pathway functioning. The whole list of genes is presented in the Additional file 3. To the best of our knowledge the present list is the most comprehensive record of genes which were biochemically validated to be related to p53 and to the key molecules of p53 pathway Mdm2, Mdmx, Puma, Slug and Chk2 till April 2011. We think that the interactions listed here are sufficiently reliable to be safely integrated into known pathways for systematic analysis.

**Patients and control samples-**

In order to evaluate the functioning of p53 pathway in leukemia, we performed bioinformatic analysis of p53 pathway-related gene expression using publicly available arrays. For this purpose we selected 4 previously published gene expression arrays performed on AML sample, which raw data was submitted to the NCBI Gene Expression Omnibus database (<http://www.ncbi.nlm.nih.gov/gds>) (GSE6891, GSE12662, GSE17061, GSE12417) [[15-18](#_ENREF_15)]. Control group that included 74 nonmalignant disorders and normal bone marrow samples (nBM) was adopted from the paper of Haferlach et al that have also deposited raw data to NCBI GEO repository (GSE13204) [[19](#_ENREF_19)]. All the arrays included in this study were conducted on the same platform of GeneChip^®^ Human Genome U133 Plus 2.0 Affymetrix Array.

All patients' samples included in this study were obtained from untreated de-novo AML patients at the time of diagnosis. The overall number of examined AML patients was 607. Both bone marrow aspirates (BM) or peripheral blood (PB) samples of AML were included, since it was shown that expression profiles from both BM and PB are highly correlated [[20](#_ENREF_20)]. Blasts and mononuclear cells were purified from bone marrow or peripheral blood aspirates of AML patients and the examined samples contained more than 70% of blast cells. Importantly, clinical data including all these parameters is available for all the patients examined in the selected arrays. Therefore, we could divide AML patients into 27 subtypes by cytogenetics, FAB and by molecular mutations. Number of patients in different AML subtypes are presented in Table 2.

**Microarray data normalization, quality control and analysis-**

Affymetrix data files of 627 patients and 74 controls in CEL format were downloaded from the NCBI Gene Expression Omnibus repository FTP site. Following the evaluation of individual arrays' quality, 20 patient arrays were excluded from the analysis. The overall quality of arrays from the 5 papers was evaluated by quality control workflow to check for gene expression value distribution consistency for individual array and found them to be satisfactory. However, we found a significant variation between the 5 different studies enrolled in our research. Study or "batch effect" is indeed expected to be present when gene expression data is combined from different studies. In order to normalize across all different arrays and minimize the batch effect, two different normalization approaches were employed: Robust Multiarray analysis (RMA) and frozen RMA (fRMA). [[21](#_ENREF_21)]. RMA quantile normalize across all array and frozen RMA computationally attempt to correct for batch effect during normalization. The results were evaluated by Principle component analysis. While potential batch differences after fRMA normalization remained in the first principle component of normalized gene expression, the effect between studies was highly attenuated in second and third principle components (data not shown). A specific linear model for identification of significant probes was developed. Since the batch effect was not entirely eliminated after fRMA, this model was also used to control for batch effect after normalization by including batch differences as covarying variables into the model to determine differential gene expression.

In Affymetrix gene expression array one gene can be represented by one or more probe sets. Identification of probe sets with significant differential expression was performed by a linear model that produced gene expression contrasts between leukemic samples and nBM. We considered a probe to be significant if it exhibited log_2_ Fold Change (log FC) value greater than 1.5 with multiple comparison adjusted p-value, using Benjamini and Hochberg method, of less than 0.01. Finally, lists of p53 pathway- related significant probes were generated for different AML patient subtypes: 12 subtypes by cytogenetics, 8 subtypes by FAB and 8 subtypes by molecular mutations. In addition, comparisons were made between patients of 3 CN-AML sub-groups and nBM. We produced a graphical representation of each list as a color-coded heat map with unsupervised clustering of probe sets. In unsupervised clustering analysis the computer groups significant probe sets based on similarity of their logFC expression. This analysis allowed us to examine the homogeneity of gene expression among different patients in the group and to visualize the differences between the controls and the AML patients.

From here onward we refer to significant probe sets as to differentially expressed genes (DEGs) in order to make a biological comprehension of the data easier. Heat maps and DEGs of different AML subtypes are available on demand.

**Data visualization by summarizing heat maps-**

We aimed to generate a "top view" for comparing alterations of p53 pathway-related gene expression between different AML groups. For this purpose we performed unsupervised hierarchical clustering using Pearson correlation coefficient as distance measurement, this time between all cytogenetic groups together based on logFC values of their DEGs. This analysis is presented as a comparative expression heat map. Similar heat maps were generated for FAB groups and molecular mutations.

**P53 pathway related gene enrichment analysis-**

We assessed the enrichment in p53 pathway related genes by measuring over-representation of p53 pathway related DEGs above the genomic background. We performed bootstrapping procedure that compared p53 pathway related DEGs to the distribution of 1153 randomly selected genes. The numbers of p53 pathway-related DEGs for each AML group is known. We determined the number of DEGs for the randomly selected genes using our linear model. The comparison was re-iterated for 10,000 times to generate the background distribution. The significance of p53 pathway-related gene enrichment was evaluated by the following equation:

$$P-val=\frac{Number of times[Number of DEG of random genes>Number of DEG from p53 ]}{Total number of iterations}$$

Over-representation of p53 pathway related genes in AML group was considered significant if p-value <0.01.

**Analysis of p53 functioning as a transcription factor –**

To evaluate whether p53 is active as a transcriptional factor in CN-AML and APL patients we analyzed the p53 pathway-related DEGs (Additional file 6) by 2 unique approaches. Individual DEGs were classified into functional outcome groups and each gene was placed in the context of p53-dependent activation or repression based on the knowledge from the literature (Additional files 8-16). Additionally, expression of our DEGs was compared to the literature-based gene expression signatures (discussed in the text, but the raw data is not shown).

1. **Real-Time PCR and IHC Methods-**

**Patients-**

This study was performed on bone marrow samples of patients diagnosed in the Department of Hematology, Hadassah Medical Center, between 1995 and 2012. All the samples were obtained from the patients prior to the initiation of therapy at the time of diagnosis. De-novo AML subtypes included: cytogenetically normal AML patients (CN-AML M0-M5, M3 excluded) and AML M3 patients at diagnosis (APL). Normal bone marrow samples from Hodgkin’s lymphoma patients without bone marrow involvement served as control for Real-Time experiments (25 for CN-AML and 34 for APL). Normal bone marrow samples of non-hematology patients (mainly with fever of unknown origin) served as control for IHC experiments (35 for both leukemia groups). All samples were initially taken for clinical needs. Use of patients’ samples was approved by Hadassah Helsinki Committee.

Karyotype of patients was examined by FISH and PML/RARA translocation was detected by the Real-Time PCR, all during the diagnostic procedure. Paired BM biopsies (used for IHC) and BM aspirates (used for RNA extraction) were taken at the same date. Clinical data of the patients examined in our study by Real-Time PCR or by IHC is presented in Additional file 2.

**PCR-**

**RNA extraction and reverse transcription-**

BM aspirates of patients were separated by Ficoll-Hypaque technique and total RNA was extracted from mononuclear fraction with TRI-reagent (MRC) and resuspended in 30µl of DEPC water. RNA was stored at -20^o^C. First-strand cDNA was prepared from 3 µl of total RNA using oligo(dT)15 primer (Promega)(for examination of Mdm2 and Mdmx) and random primer (Promega)(for examination of PCAF), dNTPs (Fermentas), M-MLV reverse transcriptase (Promega) and Ribonuclease Inhibitor (Takara).

The amount of cDNA was evaluated with GAPDH Real-time PCR reaction performed on Light Cycler Instrument (Roche Molecular Biochemicals). The average GAPDH copy number for the nBM and APL samples was greater than 1,300,000. A few samples (6/34 nBM and 4/28 APL patients) contained less than 50,000 GAPDH copies. These samples were subjected to cDNA amplification with TaqMan® PreAmp Master Mix Kit (Applied Biosystems). After the PreAmp, GAPDH copy number exceeded the average for each group. The number of GAPDH copies was checked only in some CN-AML patient samples because of their outstanding good quantity and quality of RNA.

**P53 sequencing-**

cDNA from each patient was synthesized as described above. PCR was performed in a 50μl of reaction mixture containing 16 μl of diluted cDNA, 10 μl of 5× Phusion HF buffer, 1 unit of Phusion DNA Polymerase, a proofreading polymerase with low rate of mistakes (Finnzymes, Finland), 12.5 μM of each dNTP (Fermentas) and 0.5μM each primer. The cycling conditions consisted of 30 sec at 98°C; 35 cycles of 10 s at 98°C, 20 s at 64°C, and 30 sec at 72°C; followed by a 10-min final elongation at 72°C using PTC-200 Peltier Thermal Cycler (MJ Research). The p53-specific primers provided good coverage of exons 4-10 were: forward (exon2) 5’-ggaaacattttcagacctatgga-3’, reverse (exon 11) 5’-ccctttttggacttcaggtg-3’. All PCR product quantities were measured by gel separation and at least 100 ng/ul of the product were subjected to purification and sequencing. PCR product purification was performed by the Microgen Inc (Seoul, Korea).The amplicons were subjected to direct sequencing analysis to identify mutations in the p53 exons 4-10. The PCR primers used for sequencing were: forward (exon2) 5’-gaaacattttcagacctatgga-3’, reverse (exon 11) 5’-cctttttggacttcaggtg-3’. Sequencing was performed on 3730XL DNA Analyzer (Applied Biosystems, USA) by Macrogene Inc (Seoul, Korea). Sequence data for the p53 4-10 exon region was compared with the published p53 mRNA sequence (NM_00546) using BioEdit software.

**Real time PCR-**

Real-time PCR analysis of genes was performed with commercial TaqMan® gene expression assays (Applied Biosystems): Mdm2 (Hs01066930_m1), Mdmx-fl (Hs00967242_m1). Mdm2 assay was chosen since it spans exons 2-3 that are present in most Mdm2 splicing variants. Mdmx-fl assay was chosen since it spans exons 6-7 thus detecting full length transcript only. The reactions were carried out in 15 ul of total volume using TaqMan gene expression Master mix (Applied Biosystems). Cycling conditions were according to the manufacturer’s instructions. The Tm for all genes was 60^o^C. Reactions were performed on a StepOne™ Real-Time PCR System (Applied Biosystems). In order to achieve absolute values for gene expression we constructed a calibration curve based on known copy numbers of plasmids bearing the examined genes. Mdm2 and Mdmx-fl plasmids were a kind gift of Y.Haupt, GusB and HPRT plasmids were purchased as Medium-Low abundance qPCR plasmid standards (Invitrogene). Calibration curves were prepared for each PCR run in order to control for run efficiency, but the calculation of copy number for each sample was done according to an average calibration curve constructed out of all the runs performed for the same gene. This type of calculation reduces the influence of inter-run variation on the calibration curve. Copy number value of each gene of interest was normalized to geometric mean of 2 endogenous control reference genes: GusB (Hs00939626_m1) and HPRT (Hs99999909_m1).

Results of TaqMan® gene expression assay for PCAF (Hs00908811_m1) (Applied Biosystems) was normalized to SDHA (Hs00188166_m1) and HPRT (Hs99999909_m1). The reactions were carried out in 10 ul of total volume using TaqMan gene expression Master mix (Applied Biosystems). Cycling conditions were according to the manufacturer’s instructions with Tm of 60^o^C. Reactions were performed on a ABIQuantStudio 12K Flex System (Applied Biosystems). For this gene the results were calculated using a ddCt method.

**IHC-**

**Evaluation of Ab IHC staining specificity-**

Home-made anti-Mdmx antibody (Sigma 82) and anti-Mdm2 antibody (SMP14) were a kind gift from Y.Haupt. Since those antibodies were not previously tested for IHC application, we performed a specificity examination. 293T cells were transiently transfected with Mdm2, Mdmx, or EV plasmids. Spike with GFP was used as a validation of transfection efficiency. Twenty four-48 hours after transfection some of the cells were lysed for WB analysis and the rest were embedded in the paraffin for IHC staining. Both WB and IHC showed specific staining of the transfected vs the non-transfected cells. Results are not shown.

**Immunohistochemistry staining-**

Paraffin-embedded BM biopsies were cut into 5μm pieces and adhered to the glass slide. Only freshly cut slides or slides stored at -20^o^C for up to 1 month were used for IHC staining, since the staining for most of our examined proteins decreased significantly if slides were stored at room temperature for 2 months.

Antigen retrieval was performed in a pressure cooker in citrate buffer (Real^TM^- Target Retrieval Solution, DAKO). Endogenous peroxidase was inhibited with 3% H_2_O_2_ (Hadassah pharmacy). Blocking was performed for 10 min at room temperature (Background Sniper, Biocare Medical) followed by 1 wash in washing buffer (0.1% Tween in TBS [Tris-buffered saline]). Dilutions of primary antibodies were performed in Renaissance Background Reducing Diluent (Biocare Medical). Slides were incubated in a moister chamber at 4^o^C for 16 hrs.

Antibodies used for IHC staining and the details of IHC procedure were as follows:

| **DAB time** | **Dilution** | **Raised in** | **Origin** | **Clone/catalog** | **Company** | **Antigen** |
| --- | --- | --- | --- | --- | --- | --- |
| 3 min | 1:1000 | mouse | Ascites, monoclonal | Smp14 | Home made | Mdm2 |
| 4 min | 1:75 | mouse | monoclonal | Sigma 82 | Sigma | Mdmx |
| 5 min | no dilution | mouse | hybridoma | DO1+1801 | Home made | P53 |
| 10 min | 1:100 | rabbit | polyclonal | Ab-38461 | Abcam | pChk2 |
| 10 min | 1:25 | rabbit | polyclonal | Ab-47433 | Abcam | tChk2 |
| 3 min | 1:100 | rabbit | polyclonal | C-4976 | Cell Signaling | Puma |
| 10 min | 1:100 | mouse | monoclonal | MIB-1 | Dako | KI-67 |

A polymer HRP-conjugated secondary antibody (N-Histofine® Simple Stain MAX PO (Mouse or Rabbit accordingly), Nichirei) was applied on the tissue for 30 min at room temperature. Three washes in wash buffer were followed by substrate addition (Liquid DAB+ Substrate Chromagen System, Dako). For each staining an optimum time for DAB application was determined and preserved in each staining repetition. Counter stain for the cell nucleus was done with Hematoxilin (Sigma). Slides were covered with xylene-based Histomount solution (Invitrogen). Staining without first antibody served as a negative control.

**Evaluation of immunohistochemistry staining-**

Slides were evaluated by a pathologist (T.N) and by J.A. for type of the stained cells, sub cellular localization, intensity and percent of stained cells. Assessment of staining was done for the entire examined tissue and was not based on any specific field(s). Macrophage cells were not included in this analysis. Staining evaluation was performed under high-power magnification (x40) with binocular microscope fitted with an eyepiece (x10) on Olympus BX50 microscope.

The scale for the intensity and the percentage of the stained cells was the following-

Percent- 0- 0%

0.5- 1-4%

1- 5-15%

1.5- 16-25%

2- 26-35%

2.5- 36-50%

3- 51-66%

3.5- 67-85%

4- 86-100%

Intensity- 0- negative

1- weak

2- moderate

3- strong

Multiplication of intensity score and the score for the percent of stained cells yielded a semi-quantitative IHC staining score. SQ-IHC score scale is 0-12. This method of IHC evaluation is a variation of Histoscore or H-score [[22](#_ENREF_22), [23](#_ENREF_23)].

In order to compare different staining runs for the intensity of staining, each run included a positive control. Only runs with a similar staining intensity of the positive control were included in the analysis. Otherwise, the staining was repeated. Additionally, many samples were repetitively stained in different runs in order to verify the reproducibility of staining results. Indeed, all stainings showed good reproducibility (data not shown). Moreover, the evaluation of the staining was repeated several times in order to minimize evaluation-based error.

**Statistical analysis-**

Statistical analyses were performed using SAS V9.3 statistical software (SAS Institute, Cary NC. USA). Gene expression and protein levels attained in this research have been statistically analyzed comparing nBM, CN-AML and APL groups. Protein levels were represented by 3 separate parameters (SQ, intensity, % of stained cells). We used non-parametric statistical methods for statistical analyses. Two independent continuous variables were compared with a non-parametric Wilcoxon two sample test. Correlation was assessed with Spearman’s correlation coefficient. Statistical significance is determined mostly at the 5% level of significance. To correct for multiple testing we used Hochberg's (1988) step-up Bonferroni method, when relevant.

Relative statistics was defined as the percent of subjects with overexpression of an examined protein, assuming that that the normal range of expression lies between percentiles 10 till 90 of the nBM values for that gene or protein. We determined the expression level of percentile 90 (for Mdm2, Puma, tChk2 and pChk2 proteins) or 92.5 (for Mdmx, p53 and KI-67 proteins) of nBM and assigned it as a threshold. Subsequently, we checked the number of patients that express the examined gene/protein above this threshold (all by SQ). The % of overexpressing patients was compared between groups with a Chi-Squared test or Fisher's Exact Test when relevant.

Clinical correlations-

Examination of gene expression and protein levels in this research was performed on samples of patients diagnosed in our hematology department and their clinical data was available for analysis. Two types of correlations were examined:

1. Correlation between KI-67 protein levels (by %) and the WBC data of the patients was assessed with Spearman correlation coefficient.
2. Association between SQ score of Mdmx protein and disease-free survival (DFS). DFS time was calculated from diagnosis to the date of relapse, if occurred or to the last available record if relapse did not occur. The hazard ratio was calculated from a univariate Cox regression model. The variables with significant hazard ratios were analyzed further by Kaplan-Meier survival analysis with a log-rank test that compared the survival distributions of high vs low expressing patients.

**Availability of data and materials**

Raw data of published gene expression arrays of AML samples are available in the NCBI Gene Expression Omnibus database (<http://www.ncbi.nlm.nih.gov/gds>)

Specific datasets supporting the conclusions of this article are included within the article and its additional files.

Any additional information can be provided upon request.

Reference-

1. Kannan, K., et al., *DNA microarray analysis of genes involved in p53 mediated apoptosis: activation of Apaf-1.* Oncogene, 2001. **20**(26): p. 3449-55.

2. Wang, L., et al., *Analyses of p53 target genes in the human genome by bioinformatic and microarray approaches.* J Biol Chem, 2001. **276**(47): p. 43604-10.

3. Wu, Q., et al., *Transcriptional regulation during p21WAF1/CIP1-induced apoptosis in human ovarian cancer cells.* J Biol Chem, 2002. **277**(39): p. 36329-37.

4. Spurgers, K.B., et al., *Identification of cell cycle regulatory genes as principal targets of p53-mediated transcriptional repression.* J Biol Chem, 2006. **281**(35): p. 25134-42.

5. Kho, P.S., et al., *p53-regulated transcriptional program associated with genotoxic stress-induced apoptosis.* J Biol Chem, 2004. **279**(20): p. 21183-92.

6. Heminger, K., et al., *Alterations in gene expression and sensitivity to genotoxic stress following HdmX or Hdm2 knockdown in human tumor cells harboring wild-type p53.* Aging (Albany NY), 2009. **1**(1): p. 89-108.

7. Scian, M.J., et al., *Wild-type p53 and p73 negatively regulate expression of proliferation related genes.* Oncogene, 2008. **27**(18): p. 2583-93.

8. Toledo, F. and G.M. Wahl, *Regulating the p53 pathway: in vitro hypotheses, in vivo veritas.* Nat Rev Cancer, 2006. **6**(12): p. 909-23.

9. Riley, T., et al., *Transcriptional control of human p53-regulated genes.* Nat Rev Mol Cell Biol, 2008. **9**(5): p. 402-12.

10. Nakamura, Y., *Isolation of p53-target genes and their functional analysis.* Cancer Sci, 2004. **95**(1): p. 7-11.

11. Shu, K.X., B. Li, and L.X. Wu, *The p53 network: p53 and its downstream genes.* Colloids Surf B Biointerfaces, 2007. **55**(1): p. 10-8.

12. Janicke, R.U., D. Sohn, and K. Schulze-Osthoff, *The dark side of a tumor suppressor: anti-apoptotic p53.* Cell Death Differ, 2008. **15**(6): p. 959-76.

13. Vazquez, A., et al., *The genetics of the p53 pathway, apoptosis and cancer therapy.* Nat Rev Drug Discov, 2008. **7**(12): p. 979-87.

14. Wang, B., et al., *The p53 response element and transcriptional repression.* Cell Cycle, 2010. **9**(5): p. 870-9.

15. Verhaak, R.G., et al., *Prediction of molecular subtypes in acute myeloid leukemia based on gene expression profiling.* Haematologica, 2009. **94**(1): p. 131-4.

16. Payton, J.E., et al., *High throughput digital quantification of mRNA abundance in primary human acute myeloid leukemia samples.* J Clin Invest, 2009. **119**(6): p. 1714-26.

17. Silva, F.P., et al., *Gene expression profiling of minimally differentiated acute myeloid leukemia: M0 is a distinct entity subdivided by RUNX1 mutation status.* Blood, 2009. **114**(14): p. 3001-7.

18. Metzeler, K.H., et al., *An 86-probe-set gene-expression signature predicts survival in cytogenetically normal acute myeloid leukemia.* Blood, 2008. **112**(10): p. 4193-201.

19. Haferlach, T., et al., *Clinical utility of microarray-based gene expression profiling in the diagnosis and subclassification of leukemia: report from the International Microarray Innovations in Leukemia Study Group.* J Clin Oncol, 2010. **28**(15): p. 2529-37.

20. Bullinger, L., et al., *Use of gene-expression profiling to identify prognostic subclasses in adult acute myeloid leukemia.* N Engl J Med, 2004. **350**(16): p. 1605-16.

21. McCall, M.N., B.M. Bolstad, and R.A. Irizarry, *Frozen robust multiarray analysis (fRMA).* Biostatistics, 2010. **11**(2): p. 242-53.

22. Nenutil, R., et al., *Discriminating functional and non-functional p53 in human tumours by p53 and MDM2 immunohistochemistry.* J Pathol, 2005. **207**(3): p. 251-9.

23. Taylor, C.R. and R.M. Levenson, *Quantification of immunohistochemistry--issues concerning methods, utility and semiquantitative assessment II.* Histopathology, 2006. **49**(4): p. 411-24.
