# Supplementary material for: Gene and protein analysis reveals that p53 pathway is functionally inactivated in cytogenetically normal Acute Myeloid Leukemia and Acute Promyelocytic Leukemia
Source: BMC Med Genomics. 2017 Mar 24;10:18. doi: 10.1186/s12920-017-0249-2 (PMC5423421; doi:10.1186/s12920-017-0249-2)
Supplement: Supplementary file 2 — Clinical data of the patients examined in our study by PCR and IHC. (DOCX 13 kb) [file 12920_2017_249_MOESM2_ESM.docx]

**Clinical data of the patients examined in our study by PCR and IHC**

|  | CN-AML | | APL | |
| --- | --- | --- | --- | --- |
|  | PCR | IHC | PCR | IHC |
| **BM aspirates** | 23 | 25 | 28 | 23 |
| **Pared PCR/IHC** | 12 | | 11 | |
| **Age (years, Median)** | 52.0 | 46.0 | 37.0 | 31.0 |
| **F / M (numbers)** | 10/13 | 8 / 17 | 10/18 | 13/10 |
| **WBC (Median)** | 15,200 | 19,800 | 2,200 | 1,800 |
| **Patients that had relapse** | 9 | 10 | 12 | 11 |
| **Overall survival (months)** | 11.5 | 19.0 | 77.0 | 51.0 |

The data is presented as the number of the patients in each category or as a median value for the group
