# Supplementary material for: Gene and protein analysis reveals that p53 pathway is functionally inactivated in cytogenetically normal Acute Myeloid Leukemia and Acute Promyelocytic Leukemia
Source: BMC Med Genomics. 2017 Mar 24;10:18. doi: 10.1186/s12920-017-0249-2 (PMC5423421; doi:10.1186/s12920-017-0249-2)
Supplement: Supplementary file 4 — Distribution of the genes between key proteins of p53 pathway and the overlap between them. (PPT 122 kb) [file 12920_2017_249_MOESM4_ESM.ppt]

## Slide 1
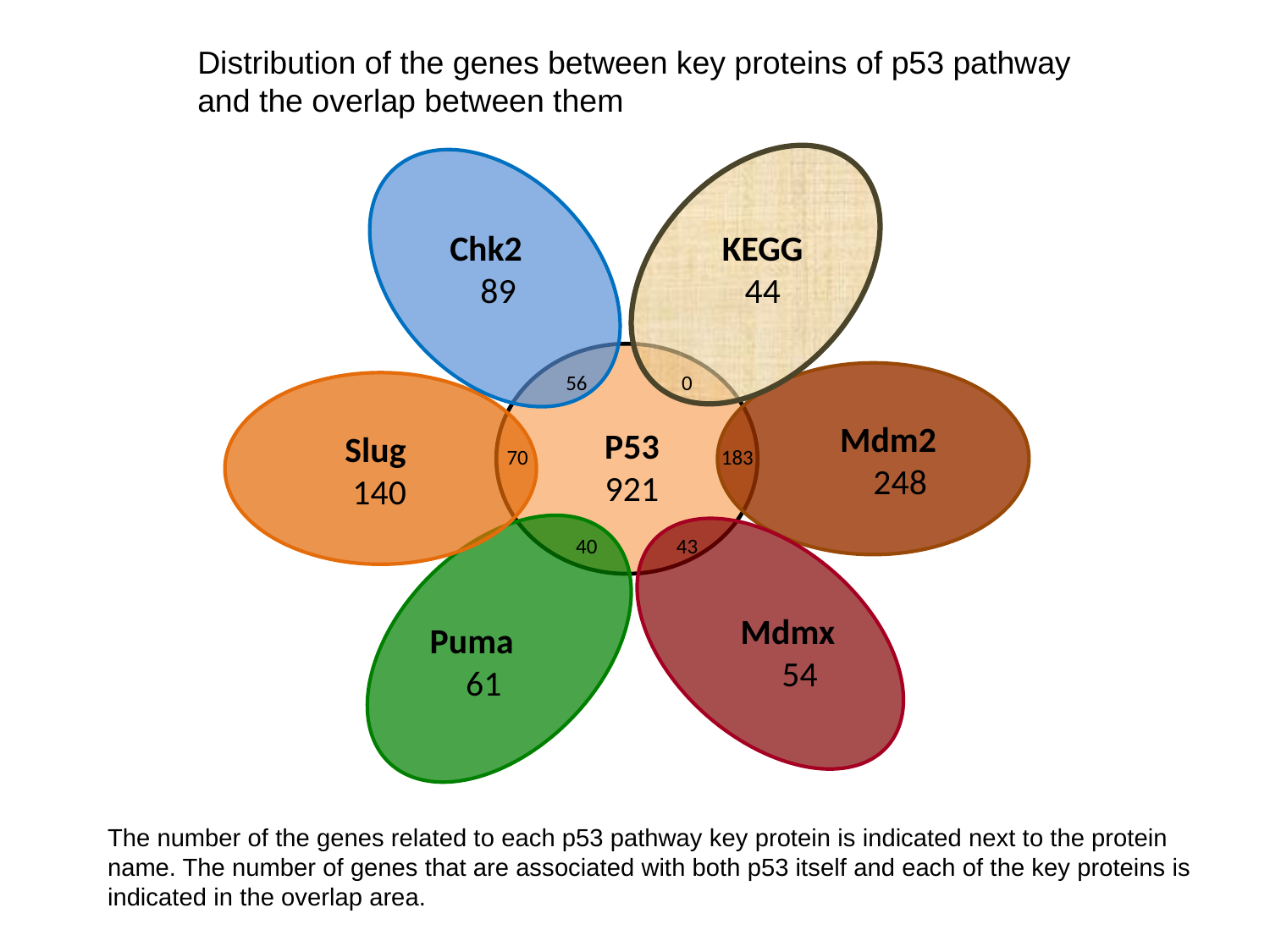

Distribution of the genes between key proteins of p53 pathway and the overlap between them
Chk2
 89
KEGG
44
56
0
Mdm2
 248
P53
921
Slug
 140
70
183
40
43
Mdmx
 54
Puma
 61
The number of the genes related to each p53 pathway key protein is indicated next to the protein name. The number of genes that are associated with both p53 itself and each of the key proteins is indicated in the overlap area.
