# Supplementary material for: Gene and protein analysis reveals that p53 pathway is functionally inactivated in cytogenetically normal Acute Myeloid Leukemia and Acute Promyelocytic Leukemia
Source: BMC Med Genomics. 2017 Mar 24;10:18. doi: 10.1186/s12920-017-0249-2 (PMC5423421; doi:10.1186/s12920-017-0249-2)
Supplement: Supplementary file 5 — Unsupervised hierarchical clustering of different AML subtypes based on their DEGs values. (PPT 599 kb) [file 12920_2017_249_MOESM5_ESM.ppt]

## Slide 1
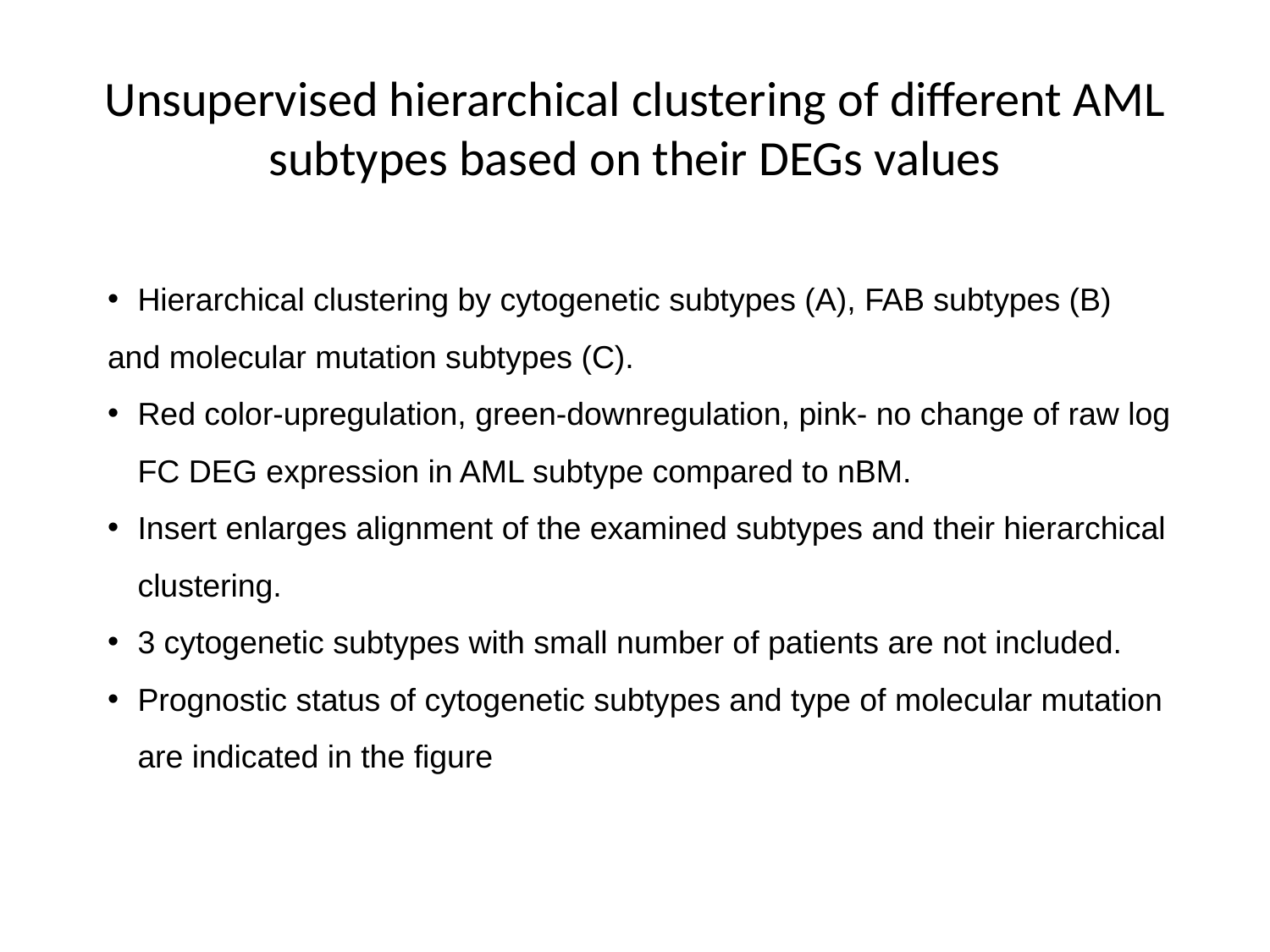

# Unsupervised hierarchical clustering of different AML subtypes based on their DEGs values
Hierarchical clustering by cytogenetic subtypes (A), FAB subtypes (B)
and molecular mutation subtypes (C).
Red color-upregulation, green-downregulation, pink- no change of raw log FC DEG expression in AML subtype compared to nBM.
Insert enlarges alignment of the examined subtypes and their hierarchical clustering.
3 cytogenetic subtypes with small number of patients are not included.
Prognostic status of cytogenetic subtypes and type of molecular mutation are indicated in the figure

## Slide 2
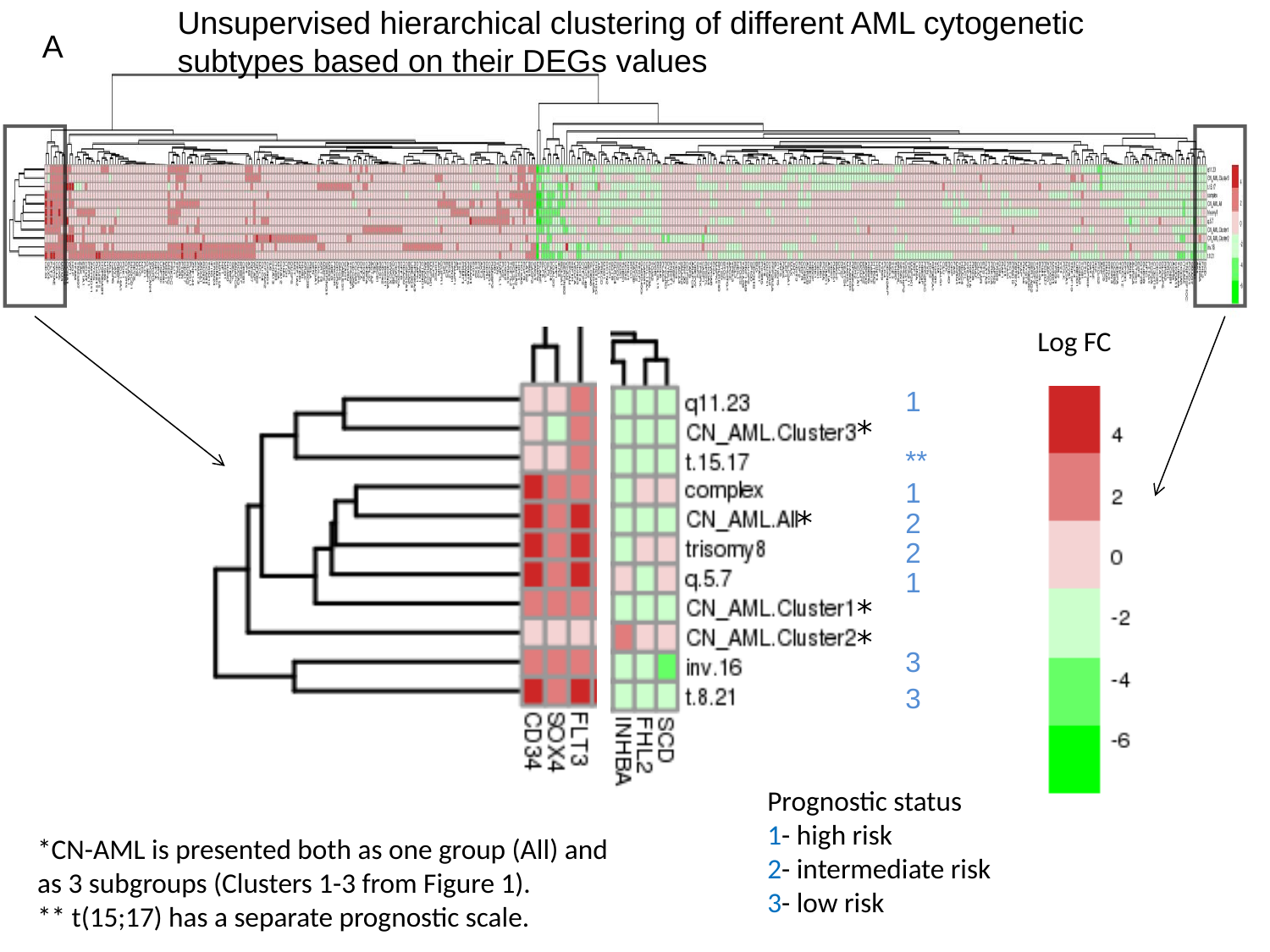

Unsupervised hierarchical clustering of different AML cytogenetic subtypes based on their DEGs values
A
Log FC
1
*
**
1
*
2
2
1
*
*
3
3
Prognostic status
1- high risk
2- intermediate risk
3- low risk
*CN-AML is presented both as one group (All) and as 3 subgroups (Clusters 1-3 from Figure 1).
** t(15;17) has a separate prognostic scale.

## Slide 3
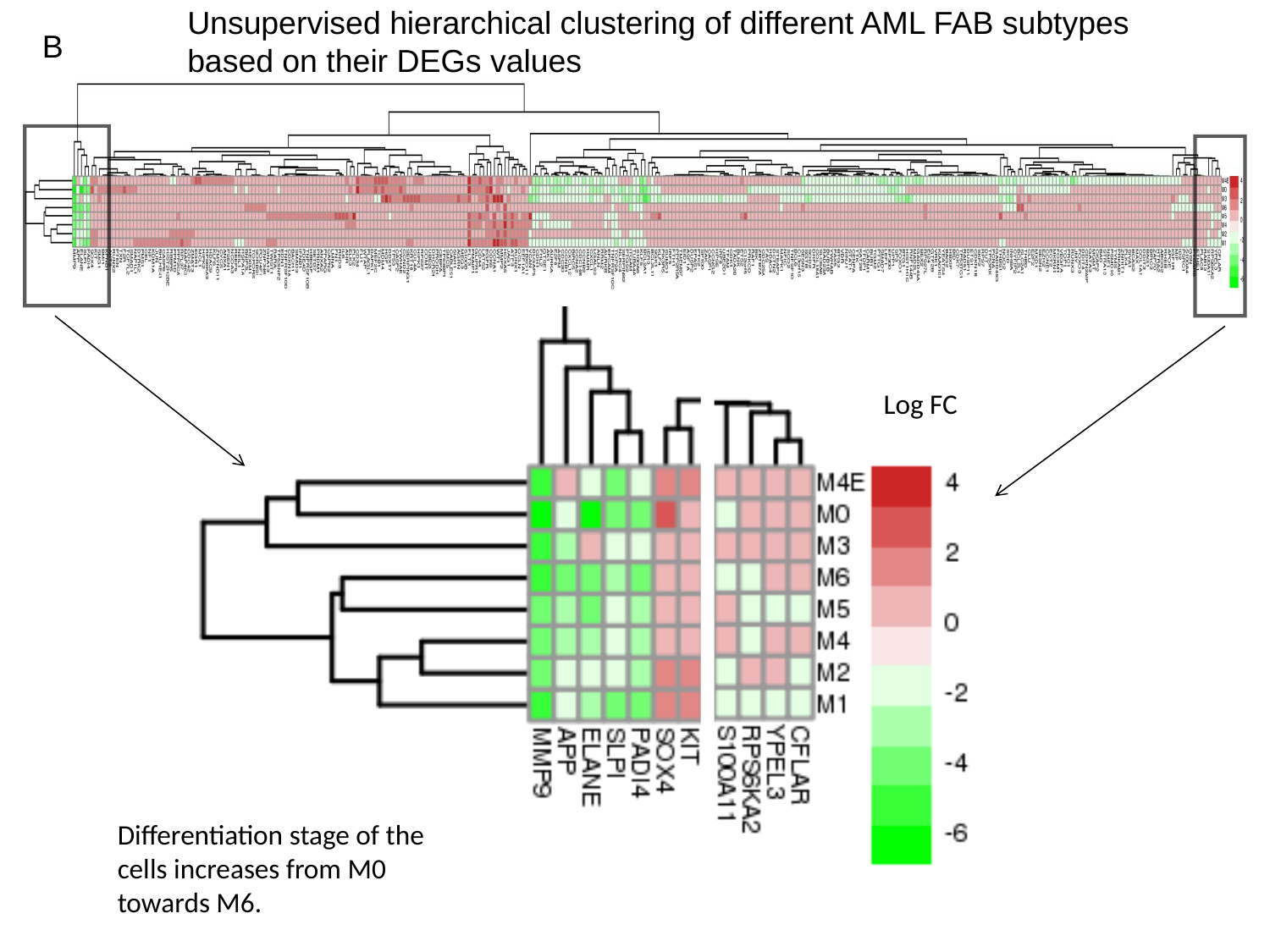

Unsupervised hierarchical clustering of different AML FAB subtypes based on their DEGs values
B
Log FC
Differentiation stage of the cells increases from M0 towards M6.

## Slide 4
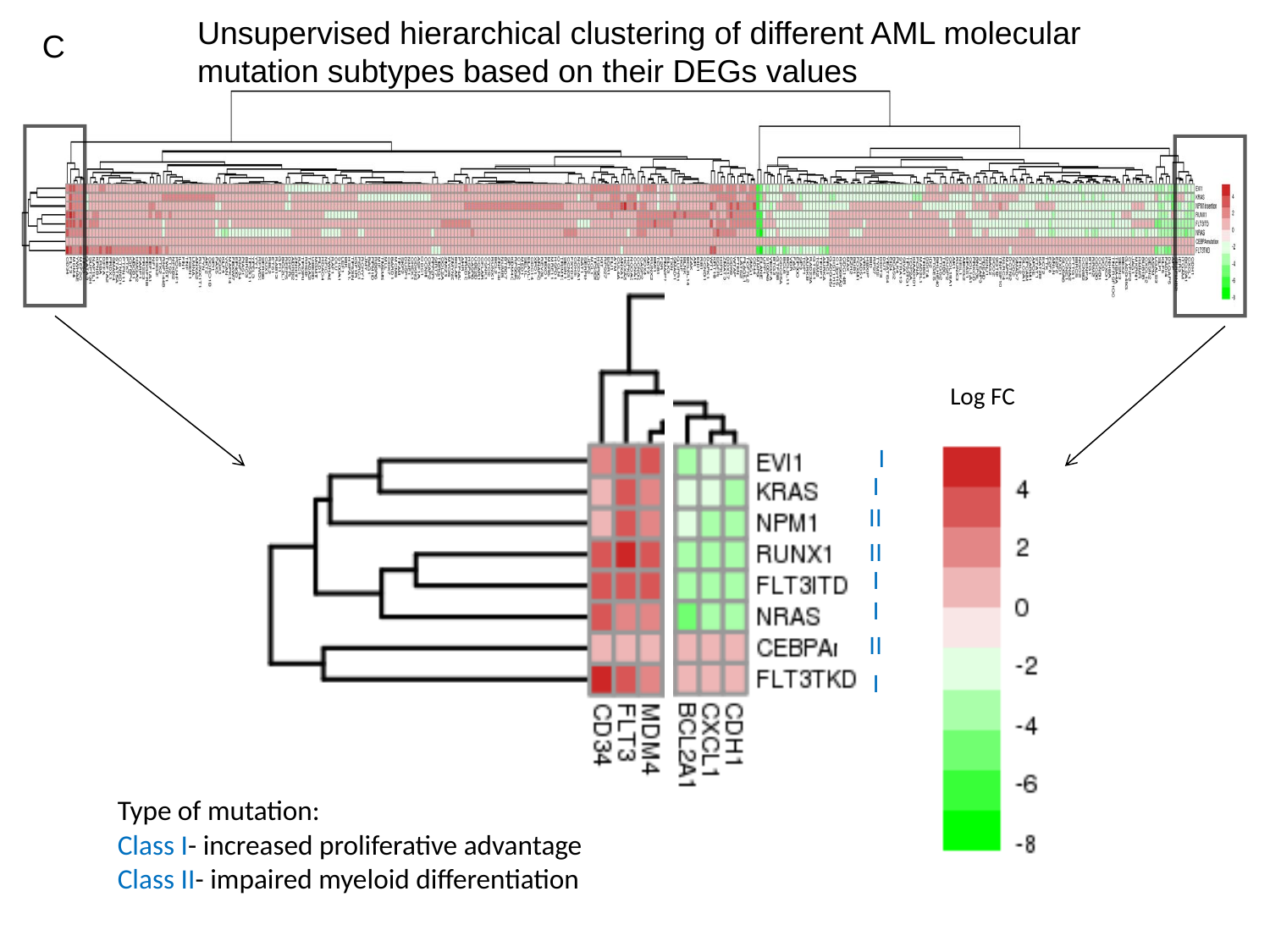

Unsupervised hierarchical clustering of different AML molecular mutation subtypes based on their DEGs values
C
Log FC
I
I
II
II
I
I
II
I
Type of mutation:
Class I- increased proliferative advantage
Class II- impaired myeloid differentiation
