# Supplementary material for: Gene and protein analysis reveals that p53 pathway is functionally inactivated in cytogenetically normal Acute Myeloid Leukemia and Acute Promyelocytic Leukemia
Source: BMC Med Genomics. 2017 Mar 24;10:18. doi: 10.1186/s12920-017-0249-2 (PMC5423421; doi:10.1186/s12920-017-0249-2)
Supplement: Supplementary file 9 — Examples of IHC staining. (PPT 2209 kb) [file 12920_2017_249_MOESM9_ESM.ppt]

## Slide 1
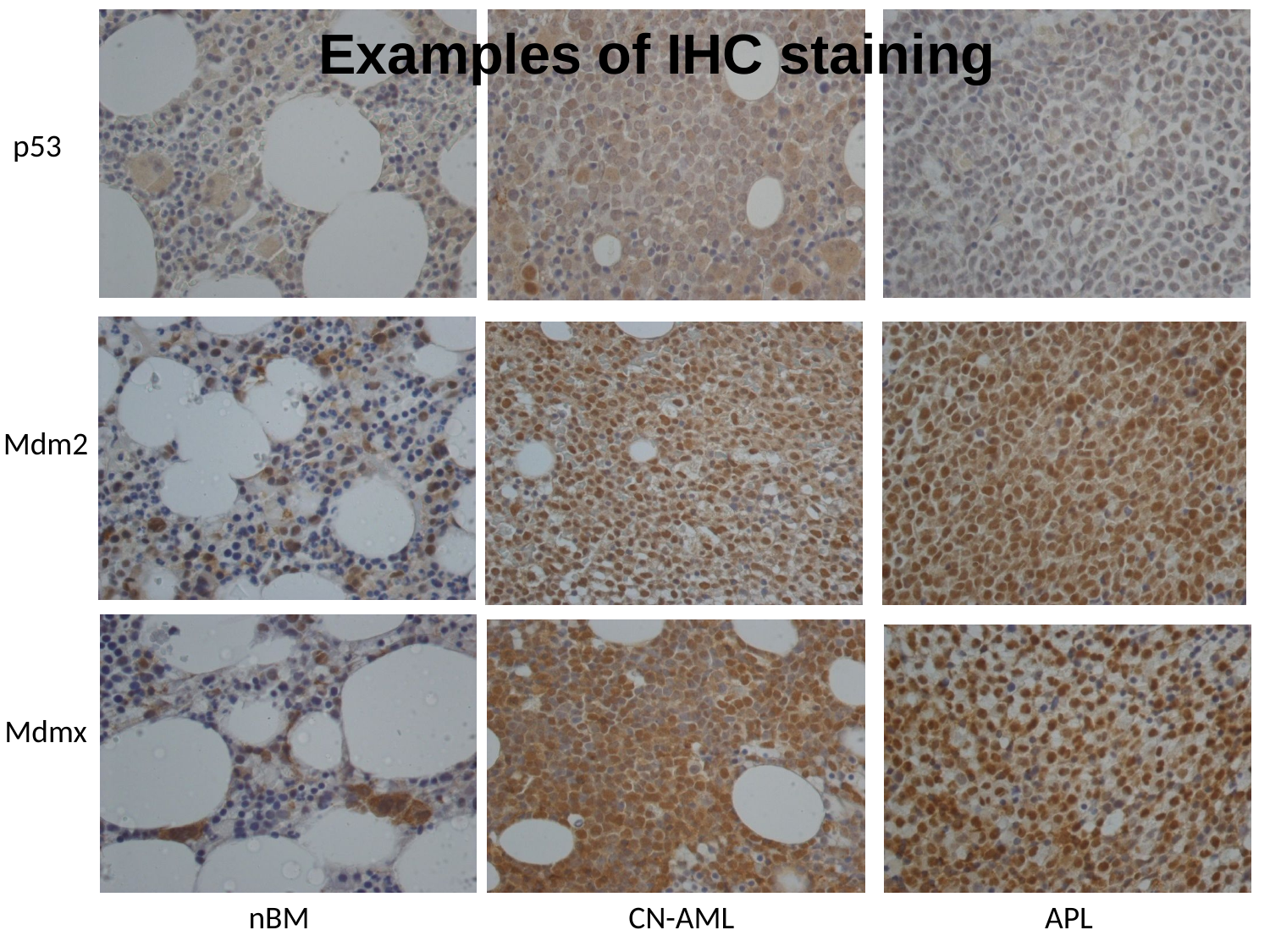

p53
Mdm2
Mdmx
nBM
CN-AML
APL
Examples of IHC staining

## Slide 2
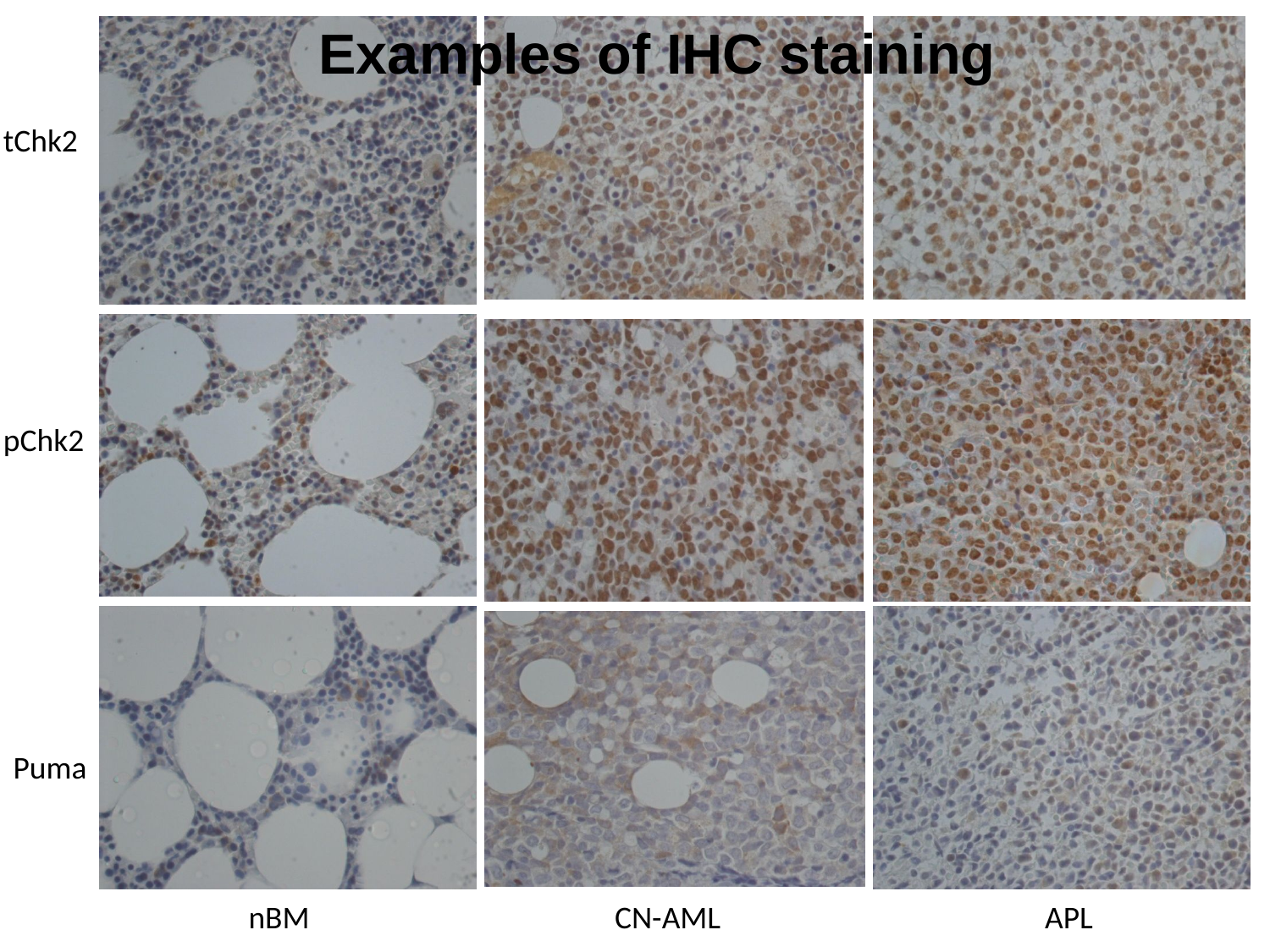

Examples of IHC staining
tChk2
pChk2
Puma
nBM
CN-AML
APL

## Slide 3
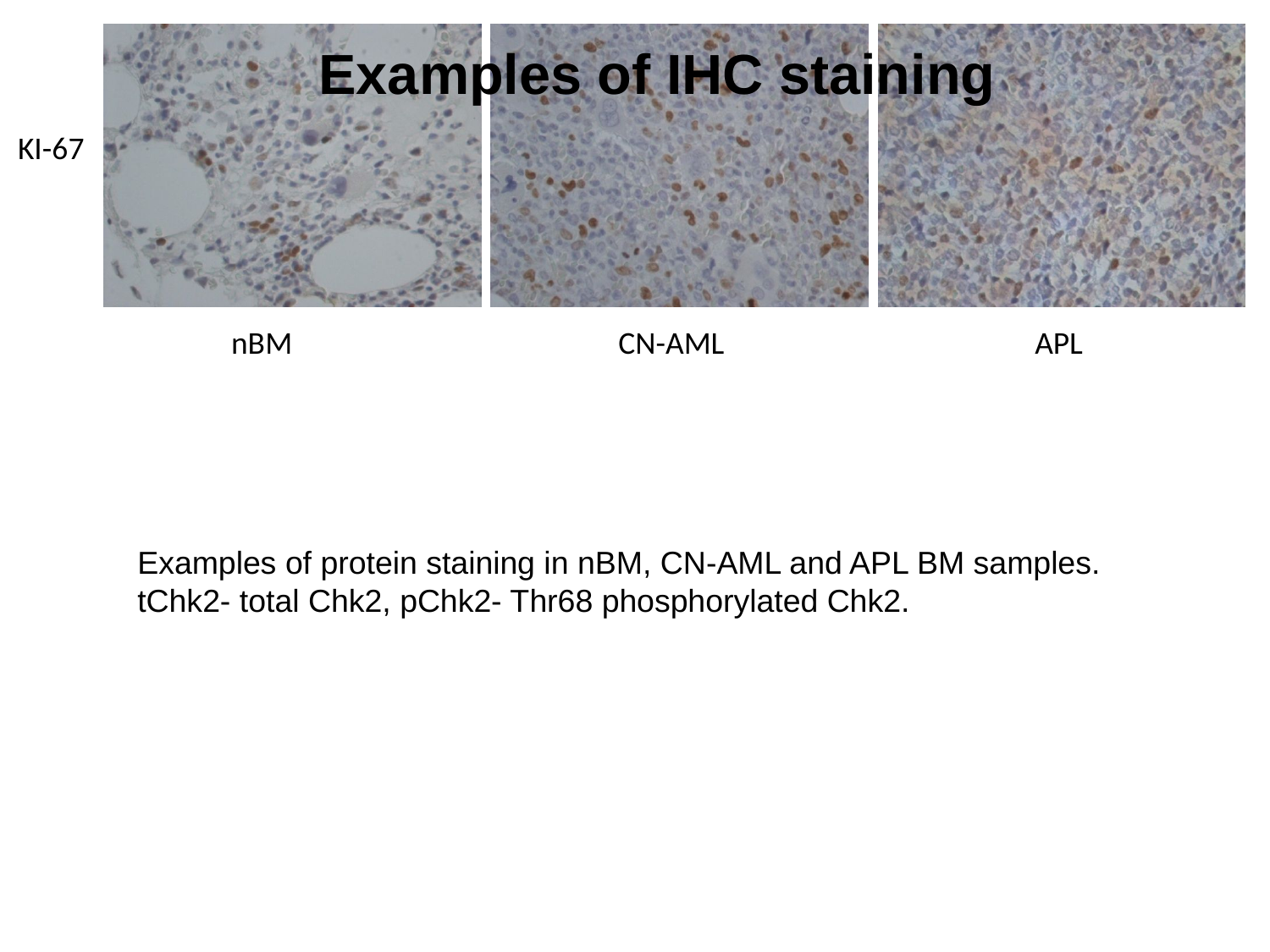

KI-67
nBM
CN-AML
APL
Examples of IHC staining
Examples of protein staining in nBM, CN-AML and APL BM samples.
tChk2- total Chk2, pChk2- Thr68 phosphorylated Chk2.
