# Supplementary material for: Gene and protein analysis reveals that p53 pathway is functionally inactivated in cytogenetically normal Acute Myeloid Leukemia and Acute Promyelocytic Leukemia
Source: BMC Med Genomics. 2017 Mar 24;10:18. doi: 10.1186/s12920-017-0249-2 (PMC5423421; doi:10.1186/s12920-017-0249-2)
Supplement: Supplementary file 17 — Protein levels by % and intensity. (PPT 394 kb) [file 12920_2017_249_MOESM17_ESM.ppt]

## Slide 1
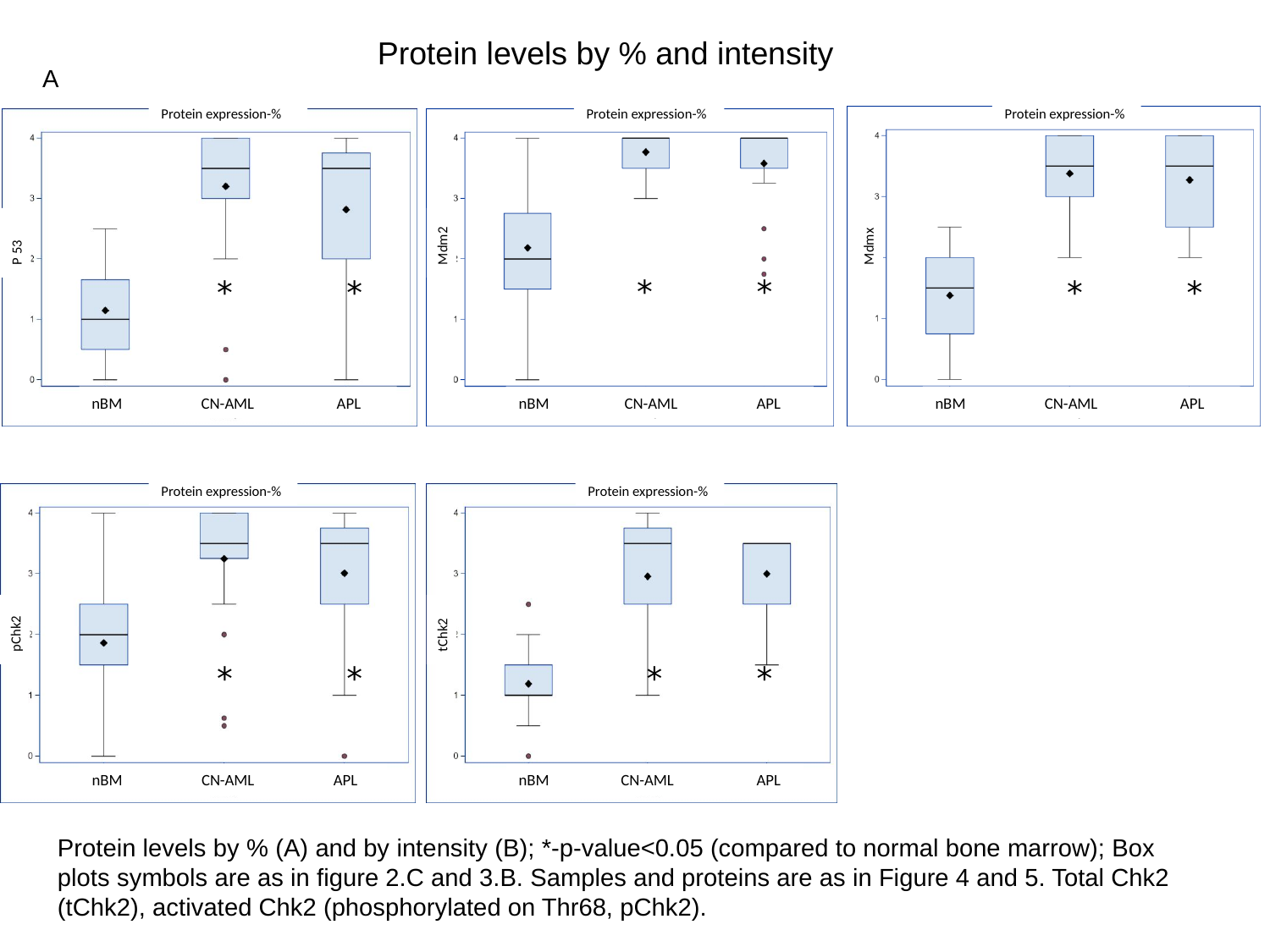

Protein levels by % and intensity
A
Protein expression-%
Protein expression-%
Protein expression-%
P 53
Mdm2
Mdmx
*
*
*
*
*
*
nBM CN-AML APL
nBM CN-AML APL
nBM CN-AML APL
Protein expression-%
Protein expression-%
pChk2
tChk2
*
*
*
*
nBM CN-AML APL
nBM CN-AML APL
Protein levels by % (A) and by intensity (B); *-p-value<0.05 (compared to normal bone marrow); Box plots symbols are as in figure 2.C and 3.B. Samples and proteins are as in Figure 4 and 5. Total Chk2 (tChk2), activated Chk2 (phosphorylated on Thr68, pChk2).

## Slide 2
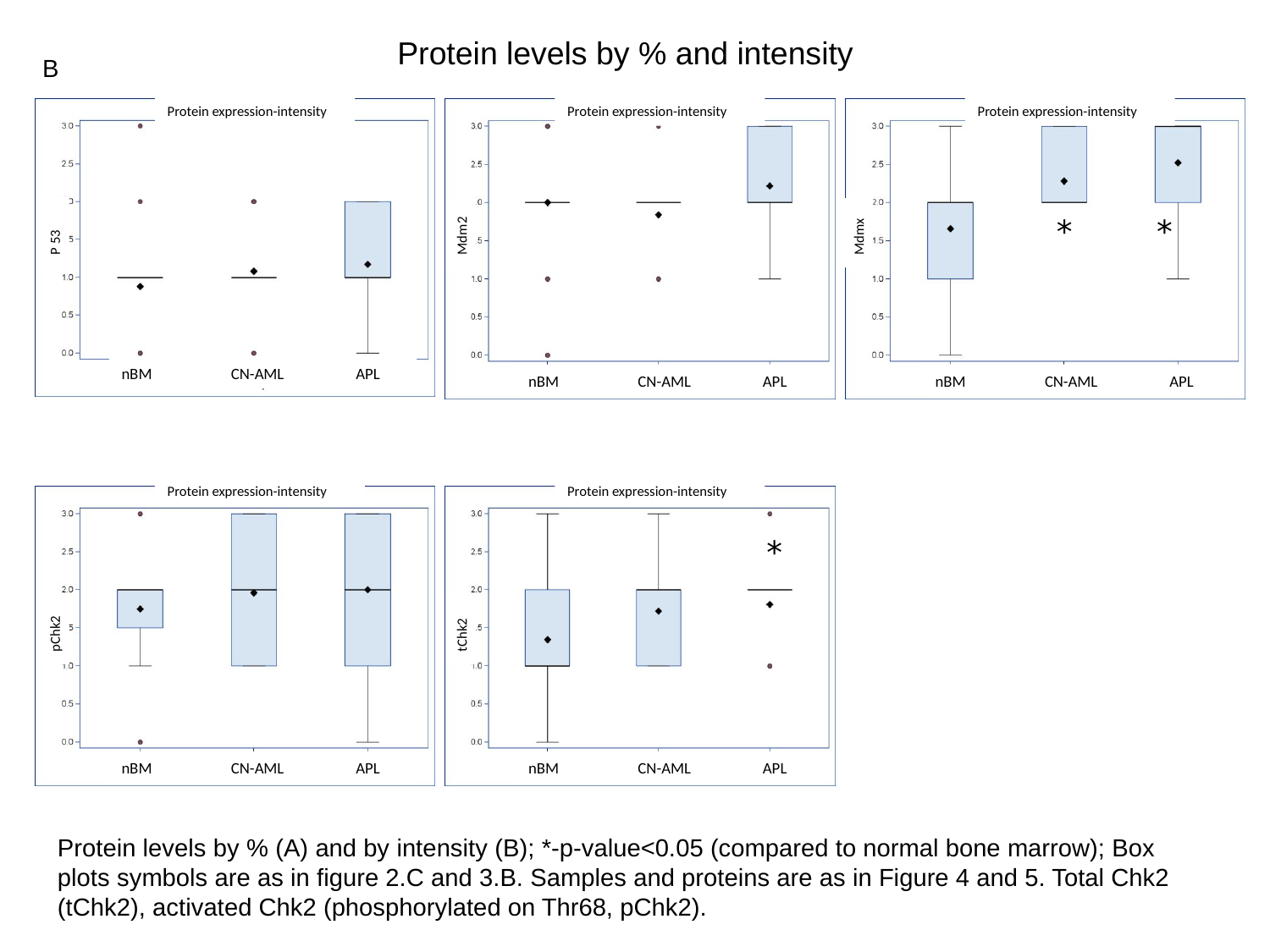

Protein levels by % and intensity
B
Protein expression-intensity
Protein expression-intensity
Protein expression-intensity
*
*
P 53
Mdm2
Mdmx
nBM CN-AML APL
nBM CN-AML APL
nBM CN-AML APL
Protein expression-intensity
Protein expression-intensity
*
pChk2
tChk2
nBM CN-AML APL
nBM CN-AML APL
Protein levels by % (A) and by intensity (B); *-p-value<0.05 (compared to normal bone marrow); Box plots symbols are as in figure 2.C and 3.B. Samples and proteins are as in Figure 4 and 5. Total Chk2 (tChk2), activated Chk2 (phosphorylated on Thr68, pChk2).
