# Supplementary material for: Gene and protein analysis reveals that p53 pathway is functionally inactivated in cytogenetically normal Acute Myeloid Leukemia and Acute Promyelocytic Leukemia
Source: BMC Med Genomics. 2017 Mar 24;10:18. doi: 10.1186/s12920-017-0249-2 (PMC5423421; doi:10.1186/s12920-017-0249-2)
Supplement: Supplementary file 18 — % of patients with protein levels above percentile 90 of nBM. (PPT 143 kb) [file 12920_2017_249_MOESM18_ESM.ppt]

## Slide 1
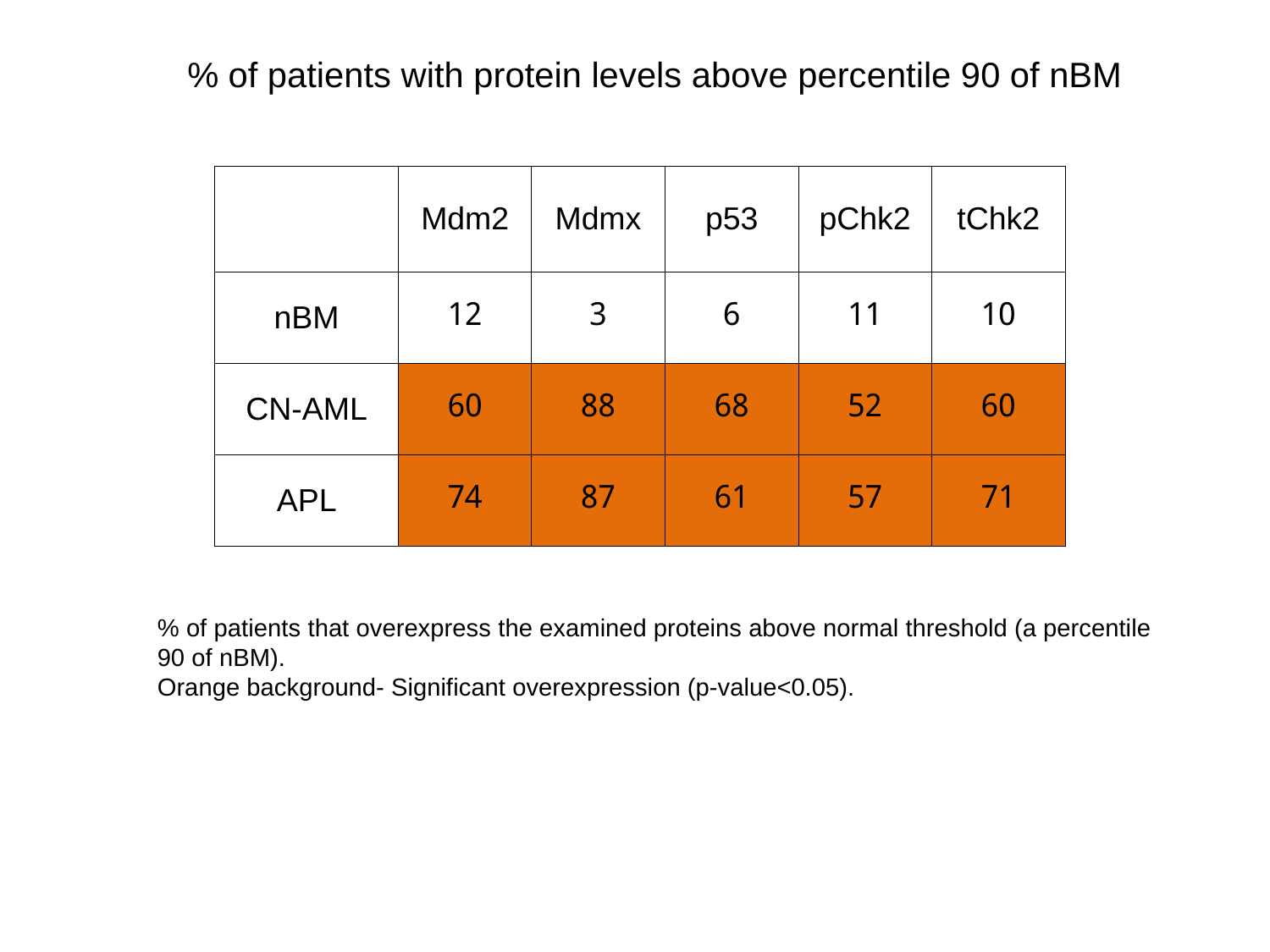

% of patients with protein levels above percentile 90 of nBM
| | Mdm2 | Mdmx | p53 | pChk2 | tChk2 |
| --- | --- | --- | --- | --- | --- |
| nBM | 12 | 3 | 6 | 11 | 10 |
| CN-AML | 60 | 88 | 68 | 52 | 60 |
| APL | 74 | 87 | 61 | 57 | 71 |
% of patients that overexpress the examined proteins above normal threshold (a percentile 90 of nBM).
Orange background- Significant overexpression (p-value<0.05).
